# The Burden of Seasonal Influenza and Its Potential Complications Among Older Japanese Adults: A Real‐World Database Study

**DOI:** 10.1111/irv.70032

**Published:** 2024-11-12

**Authors:** Takeshi Arashiro, Yuki Tajima, Yohei Ban, Matthew M. Loiacono, Masayo Ideguchi, Caroline de Courville

**Affiliations:** ^1^ Vaccines Medical Sanofi K.K. Tokyo Japan; ^2^ Medical Affairs Real World Evidence Generation Partnering Sanofi K.K. Tokyo Japan; ^3^ Real‐World Evidence Department INTAGE Healthcare, Inc. Tokyo Japan; ^4^ Medical Evidence Generation Sanofi Swiftwater Pennsylvania USA; ^5^ Health Economics and Value Assessment, Market Access Sanofi K.K. Tokyo Japan; ^6^ Health Economics and Value Assessment Sanofi Lyon France

**Keywords:** aged, healthcare costs, hospitalization, human, influenza, Japan, mortality

## Abstract

**Background:**

Seasonal influenza may lead to severe complications, including respiratory and cardiovascular disease, that result in considerable healthcare resource utilization (HCRU) and mortality, particularly in older individuals. This real‐world study assessed the burden of influenza and its potential complications in older Japanese adults.

**Methods:**

This retrospective claims database analysis (April 2015 to June 2019) included insured individuals aged ≥ 60 years at the start of four consecutive influenza seasons in Japan (October 1 to April 30). The primary endpoint was the incidence of influenza‐related emergency room (ER) and outpatient visits, the incidence of hospitalizations, the probability of 30‐day inpatient mortality, and 60‐day medical costs of influenza or its potential complications.

**Results:**

Of 8,974,708 individuals (43.2% male, mean ± standard deviation age 73.8 ± 8.9 years), 370,430 (4.13%) were diagnosed with influenza. Overall, 17.18 (95% confidence interval [CI] 16.32–18.04) and 3893.53 (3880.87–3906.19) per 100,000 individuals had influenza‐related ER and outpatient visits, respectively, and 181.50 (178.71–184.28) per 100,000 individuals were hospitalized for influenza. The incidence of influenza‐related ER visits and hospitalizations for influenza or potential complications and the probability of 30‐day inpatient mortality increased with age.

**Conclusions:**

Seasonal influenza and its potential complications represent a substantial burden that increases with age in Japanese individuals.

## 1 Introduction

Seasonal influenza is a contagious acute respiratory viral infection that is mainly caused by influenza A (e.g., H1N1 and H3N2 strains) and influenza B (e.g., Victoria strains), affecting approximately 1 billion people annually worldwide [1, 2]. It is predominantly a winter epidemic in Japan [3]. Influenza infection may lead to severe complications across multiple organ systems, including an increased risk of myocardial infarction and stroke [4], glycemic events, pneumonia, and ischemic heart disease in individuals with type 2 diabetes mellitus [5], and leads to between 290,000 and 650,000 respiratory deaths worldwide each year [1].

The World Health Organization (WHO) recommends implementation of seasonal influenza vaccination programs in all countries, with older adults (including those living in long‐term care facilities) among the targeted groups considered for immunization [6]. Currently, the vaccination policy in Japan recommends routine seasonal influenza vaccination for people aged ≥ 65 years and in high‐risk individuals aged ≥ 60 years [7]. Given that the proportion of individuals aged ≥ 65 years in Japan has been predicted to increase from 28.4% of the population in 2019 to 35.3% in 2040 [8], effective implementation of this vaccination policy will be essential to reduce the burden of influenza in these older individuals.

The progression and severity of influenza symptoms may vary between people; however, the risk of influenza‐related hospitalization and mortality is highest in older individuals and those with chronic comorbidities (e.g., diabetes mellitus, heart disease, asthma, and chronic kidney disease) [9]. Further, because of immunosenescence (i.e., the progressive decrease in natural systemic immunity against infectious diseases with age) [10], older individuals have an increased risk of serious health complications [11] and often experience functional decline following hospitalization for influenza [12, 13].

The increased risk of serious influenza and its potential complications in older individuals translates into increased healthcare resource utilization (HCRU) and economic costs [14, 15]. Globally, individuals aged ≥ 70 years had the highest rates of mortality due to influenza lower respiratory tract infections (16.4 deaths per 100,000) in 2017, as well as the greatest proportion of influenza cases hospitalized (approximately 40%–50%) [16]. Further, data on the increased risk of influenza‐related hospitalizations among older adults have been reported in the United States (US) and Europe [17, 18]. In the 2022–2023 season in the US, 45.2% of all laboratory‐confirmed influenza hospitalizations were among individuals aged ≥ 65 years [17]. In Europe, 88% of seasonal influenza‐related deaths were among individuals aged ≥ 65 years, with the mortality rate during 2002–2011 being approximately 35 times higher in these individuals than in those aged < 65 years (0.8 vs. 29.0 per 100,000, respectively) [18].

With regard to the economic burden, the largest proportion of the direct costs of influenza in the US have been attributed to individuals aged ≥ 65 years, primarily because of influenza‐related hospitalizations [19]. Further, the total medical costs associated with influenza in a German study were higher in adults aged ≥ 60 years than in those aged < 60 years, mainly because of higher hospitalization costs for influenza and potential complications [20].

Influenza also represents a significant burden in Japan, particularly among older individuals [21, 22]. Longitudinal surveillance of influenza found an increasing incidence rate of influenza over time in Japanese individuals aged ≥ 60 years, from 0.02 per year in 2005–2006 to 0.07 per year in 2015–2016 [22]. In a database study of 111 million insured Japanese people from September 2017 to August 2020, 31 million influenza cases required general practitioner visits, 512,000 required hospitalization, and there were 28,000 influenza‐related deaths [23]. In this study, influenza‐related hospitalization and mortality rates tended to be higher in older individuals than in younger or middle‐aged individuals [23]. Furthermore, a retrospective study showed that the economic burden of hospitalization for influenza was high, with > 60% of hospitalized individuals being aged ≥ 65 years [24]. However, data on the overall burden of seasonal influenza and its related diseases in Japan, particularly with regard to the associated HCRU (including outpatient and emergency room [ER] visits), mortality, and medical costs in older individuals, are scarce.

The primary objective of this real‐world database study was to assess the burden of influenza among older people in Japan in terms of (1) ER and outpatient visits for influenza; (2) hospitalizations for influenza and its potential complications; (3) all‐cause hospitalizations within 28 days of the first influenza diagnosis; (4) the probability of 30‐day inpatient mortality due to influenza and its potential complications; and (5) average medical costs associated with HCRU for influenza and its potential complications. This study also assessed the overall probability of influenza‐related mortality and all outcomes according to age group.

## 2 Methods

### 2.1 Study Design

This retrospective cohort study used data extracted from an insurance claims database provided by DeSC Healthcare, Inc. (Tokyo, Japan). The DeSC database contains deidentified claims and HCRU data, including all medical diagnoses, prescribed medications, reimbursed costs for examinations and procedures, and data from annual health visits, from individuals insured by three main insurance associations in Japan (Supplementary Methods): Kempo (the Health Insurance Association for employees of large companies), Kokuho (the National Health Insurance Association for self‐employed or retired individuals), and Koki Koreisha Iryo Seido (the Advanced Elderly Medical Service System for individuals aged ≥ 75 years) [25], of whom 5.68% were aged ≥ 60 years (Table S1). In the database, International Classification of Disease–10^th^ revision (ICD‐10) diagnostic codes are used for disease names, Anatomical Therapeutic Chemical classification codes are used for drug names, and medical costs per individual correspond to the amount billed paid by the insurance association. This study used claims data from April 1, 2015, to June 30, 2019, including four influenza seasons (i.e., October 1 to April 30 of the following year) prior to the coronavirus disease 2019 pandemic (Figure S1). In addition, April 1 to September 30 was set as the baseline period (i.e., before the start of the influenza season) for each influenza season.

This noninterventional study was conducted in accordance with the Ethical Guidelines for Research in the Life Sciences and Medical Sciences on Human Subjects. The study used deidentified data from an insurance claims database; therefore, the requirement for informed consent was waived because of the anonymous nature of the database. The ethics committee of SOUKEN Co., Ltd. reviewed and approved the study prior to study initiation.

### 2.2 Study Population

The study population consisted of all eligible insured individuals in the database, from whom only individuals with mortality data available were included in the mortality analysis. Eligible individuals were those aged ≥ 60 years as of October 1 in each influenza season (i.e., October 1 to April 30) and who had continuous data available from the prior 6‐month baseline period (i.e., April 1 to September 30; Figure S1). Individuals from each influenza season were assessed for inclusion, with data obtained from the baseline period of each season used to determine study eligibility. To assess the burden of influenza specifically during each season, those who were diagnosed with influenza (ICD‐10 codes J09–J11, excluding suspected influenza) or prescribed an anti‐influenza drug (Table S2) during the baseline period were excluded. Individuals who were diagnosed with influenza during the influenza season but had their first influenza‐related medical examination during the preceding baseline period were also excluded.

### 2.3 Objectives and Outcomes

The overall study objective was to assess the burden of influenza and its potential complications among older people in Japan. The primary endpoint comprised the following five outcomes: (1) ER and outpatient visits for influenza; (2) hospitalizations for influenza and its potential complications (i.e., influenza broadly defined [influenza or pneumonia], respiratory disease, or respiratory or cardiovascular disease, as described in the Supplementary Methods); (3) all‐cause hospitalizations within 28 days of the first influenza diagnosis; (4) the probability of inpatient mortality within 30 days of admission due to influenza and its potential complications; and (5) the average direct medical costs per hospitalization (within 60 days of influenza‐related admission), ER visit, and outpatient visits. Outcomes (1) to (3) were presented as the absolute number and incidence proportion per 100,000 individuals per season (average across four seasons). If an individual had multiple influenza‐related ER visits or outpatient visits or was hospitalized multiple times for influenza or its potential complications within the influenza season, these were considered to be the same HCRU event. Secondary endpoints were the overall probability of mortality among all individuals with influenza within 30 days of symptom onset, as well as all outcomes according to age group at the start of the influenza season (≥ 60 to < 65, ≥ 65 to < 75, or ≥ 75 years). Other outcomes included the distribution of the duration of hospitalization for influenza and its potential complications and assessment of HCRU, medical costs, and mortality outcomes by influenza season and in individuals aged ≥ 60 to < 65 years with or without ≥ 1 comorbidity. Definitions of all outcomes are provided in Table S3.

In sensitivity analyses, the 30‐ and 90‐day medical costs and the probability of 60‐ and 90‐day inpatient mortality probability were assessed.

All outcomes, except for mortality, were analyzed in the total study population. The probability of mortality was assessed among those with available mortality data.

### 2.4 Statistical Analysis

Data were presented using descriptive statistics, with continuous variables presented as mean ± standard deviation (SD) or median with first and third quartiles (Q1, Q3) and categorical variables presented as individual number and percentage.

For the primary and secondary endpoint outcomes, the average incidence proportion per 100,000 individuals per season (across all four seasons), the probability of 30‐day inpatient mortality, and the overall probability of mortality within 30 days of symptom onset were calculated as described in the Supplementary Methods. The 95% confidence interval of Wald was also calculated for each of these outcomes.

For the direct medical costs due to influenza or its potential complications (primary endpoint (5)), the summary statistics (mean ± SD) for the total medical costs (including the sum of all medical practice and pharmaceutical costs) per individual were calculated in Japanese Yen (¥) for all outpatient and ER visits due to influenza and within 60 days of admission for hospitalizations due to influenza or its potential complications.

Statistical analyses were conducted using SAS, version 9.4 (SAS Institute, Cary, NC, USA).

## 3 Results

### 3.1 Study Population

**FIGURE 1:**
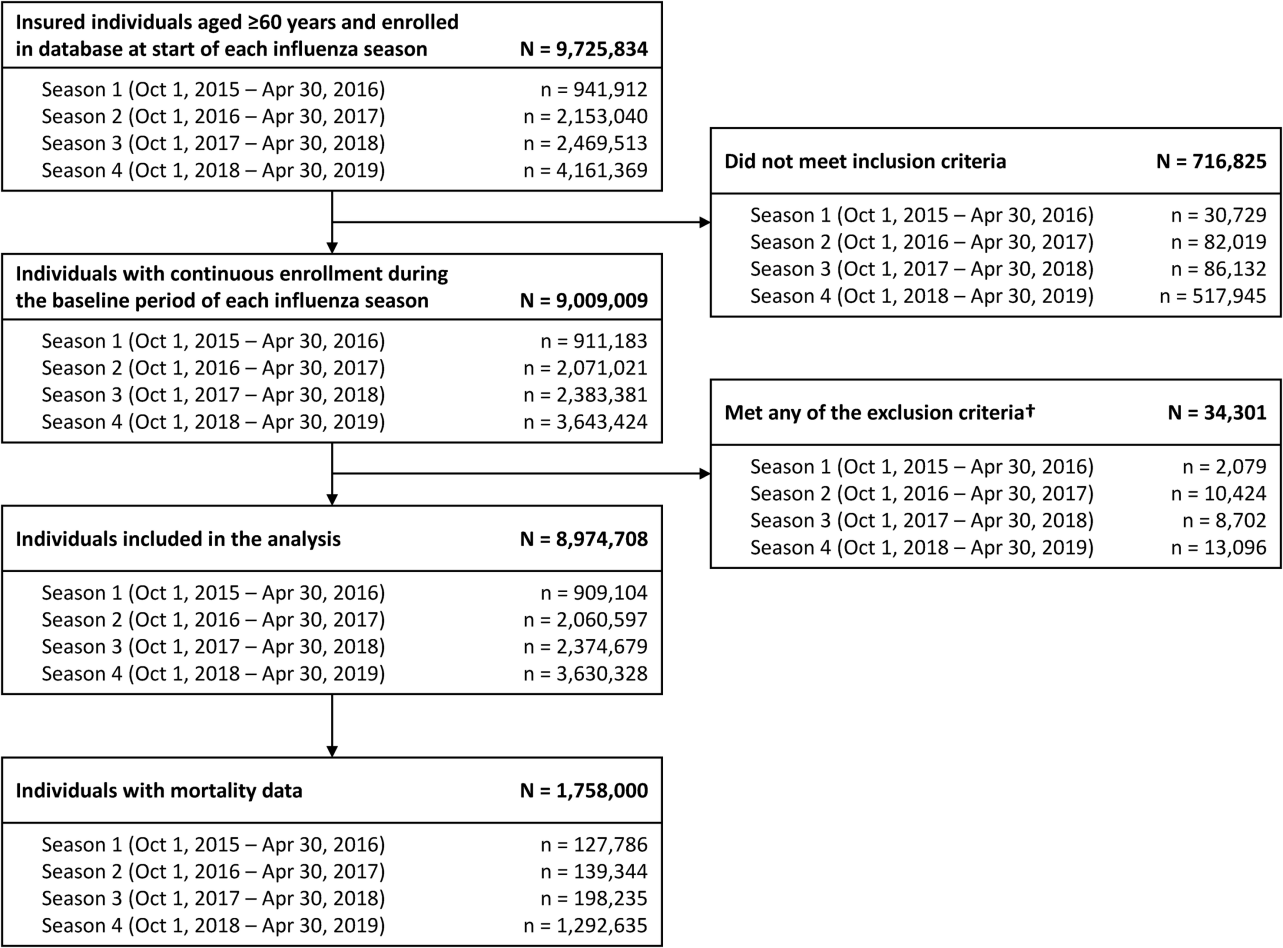
Study population flow. †Individuals who were diagnosed with influenza (defined as International Classification of Disease–10^th^ revision diagnostic codes J09–J11; excluding suspected influenza) before the start of each influenza season or were prescribed an anti‐influenza drug during the same period.

Across all four influenza seasons, 9,725,834 individuals aged ≥ 60 years were enrolled in the database. Of these, 8,974,708 individuals met the inclusion criteria and were included in the total study population; mortality data were available for 1,758,000 individuals and 370,430 were diagnosed with influenza (Figure 1).

In the total study population, 43.2% were male and the mean ± SD age was 73.8 ± 8.9 years (Table 1). The most common comorbidities were diabetes mellitus (27.6%), cancer (15.6%), and ischemic heart disease (14.5%). At least one comorbidity was present in 51.2% of all individuals, 33.9% of those aged ≥ 60 to < 65 years, 44.2% of those aged ≥ 65 to < 75 years, and 62.4% of those aged ≥ 75 years (Table 1). The baseline characteristics of individuals with mortality data and individuals diagnosed with influenza are shown in Tables S4 and S5, respectively.

**TABLE 1:** Baseline characteristics of the total study population and age groups.

	Total study population *N* = 8,974,708	Age group, years		
		≥ 60 to < 65 *n* = 1,299,552	≥ 65 to < 75 *n* = 3,501,469	≥ 75 *n* = 4,173,687
Male sex, *n* (%)	3,874,348 (43.2)	567,267 (43.7)	1,638,222 (46.8)	1,668,859 (40.0)
Age, mean ± SD, years	73.8 ± 8.9	—	—	—
Type of insurance, *n* (%)				
Kempo^a^	254,777 (2.8)	122,080 (9.4)	132,697 (3.8)	0
Kokuho^b^	4,410,795 (49.1)	1,177,472 (90.6)	3,233,323 (92.3)	0
Koki Koreisha Iryo Seido^c^	4,309,136 (48.0)	0	135,449 (3.9)	4,173,687 (100.0)
Comorbidities, n (%)				
≥ 1 comorbidity	4,592,655 (51.2)	440,350 (33.9)	41,549,004 (44.2)	2,603,301 (62.4)
Diabetes mellitus	2,479,304 (27.6)	233,836 (18.0)	884,149 (25.3)	1,361,319 (32.6)
Cancer	1,403,497 (15.6)	133,087 (10.2)	485,661 (13.9)	784,749 (18.8)
Ischemic heart disease	1,299,295 (14.5)	75,628 (5.8)	341,040 (9.7)	882,627 (21.1)
Asthma	775,347 (8.6)	82,977 (6.4)	256,458 (7.3)	435,912 (10.4)
Atrial fibrillation	462,748 (5.2)	21,027 (1.6)	110,758 (3.2)	330,963 (7.9)
Congestive heart failure	389,307 (4.3)	16,867 (1.3)	72,825 (2.1)	299,615 (7.2)
Chronic renal failure	325,274 (3.6)	19,672 (1.5)	78,633 (2.2)	226,969 (5.4)
COPD	236,821 (2.6)	11,528 (0.9)	64,061 (1.8)	161,232 (3.9)
Pneumonia	228,878 (2.6)	13,216 (1.0)	51,575 (1.5)	164,087 (3.9)
Parkinson's disease	172,444 (1.9)	18,932 (1.5)	45,873 (1.3)	107,639 (2.6)
Acute respiratory failure	67,498 (0.8)	3381 (0.3)	14,129 (0.4)	49,988 (1.2)
Acute renal failure	17,368 (0.2)	1083 (0.1)	4135 (0.1)	12,150 (0.3)
HIV/AIDS	1462 (0.02)	308 (0.02)	640 (0.02)	514 (0.01)

Abbreviations: AIDS, autoimmune deficiency syndrome; COPD, chronic obstructive pulmonary disease; HIV, human immunodeficiency virus; SD, standard deviation.

^a^ The Health Insurance Association for employees of large companies.

^b^ The National Health Insurance Association for self‐employed or retired individuals.

^c^ The Advanced Elderly Medical Service System for individuals aged ≥ 75 years.

### 3.2 Healthcare Resource Utilization

Across the four influenza seasons, the incidence proportion of ER visits and outpatient visits for influenza per 100,000 individuals per season was 17.18 and 3893.53, respectively (Table 2). The incidence proportion of hospitalizations per 100,000 individuals per season was 181.50 for influenza, 1242.50 for influenza broadly defined, 2176.49 for respiratory disease, 4700.63 for respiratory or cardiovascular disease, and 307.39 for all‐cause hospitalizations within 28 days of the first influenza diagnosis (Table 2).

**TABLE 2:** Average healthcare resource utilization events per season for influenza or its potential complications (across all four seasons).

	Total study population *N* = 8,974,708	Age group, years		
		≥ 60 to < 65 *n* = 1,299,552	≥ 65 to < 75 *n* = 3,501,469	≥ 75 *n* = 4,173,687
ER visits for influenza^a^ ^,^ ^b^				
Absolute number of ER visits	1542	63	220	1259
Incidence proportion per 100,000 individuals	17.18	4.85	6.28	30.17
[95% CI]	[16.32–18.04]	[3.65–6.04]	[5.45–7.11]	[28.50–31.83]
Outpatient visits for influenza^a^ ^,^ ^b^				
Absolute number of outpatient visits	349,433	61,341	138,327	149,765
Incidence proportion per 100,000 individuals	3893.53	4720.17	3950.54	3588.31
[95% CI]	[3880.87–3906.19]	[4683.70–4756.63]	[3930.14–3970.95]	[3570.47–3606.16]
Hospitalizations for influenza^a^ ^,^ ^b^				
Absolute number of hospitalizations	16,289	504	2324	13,461
Incidence proportion per 100,000 individuals	181.50	38.78	66.37	322.52
[95% CI]	[178.71–184.28]	[35.40–42.17]	[63.67–69.07]	[317.08–327.96]
Hospitalizations for influenza broadly defined (influenza or pneumonia)^b^ ^,^ ^c^				
Absolute number of hospitalizations	111,511	3142	16,817	91,552
Incidence proportion per 100,000 individuals	1242.50	241.78	480.28	2193.55
[95% CI]	[1235.26–1249.75]	[233.33–250.22]	[473.04–487.53]	[2179.50–2207.60]
Hospitalizations for respiratory disease^b^ ^,^ ^d^				
Absolute number of hospitalizations	195,334	6777	34,749	153,808
Incidence proportion per 100,000 individuals	2176.49	521.49	992.41	3685.18
[95% CI]	[2166.95–2186.04]	[509.10–533.87]	[982.03–1002.79]	[3667.11–3703.26]
Hospitalizations for respiratory or cardiovascular disease^b^ ^,^ ^e^				
Absolute number of hospitalizations	421,868	17,653	84,539	319,676
Incidence proportion per 100,000 individuals	4700.63	1358.39	2414.39	7659.32
[95% CI]	[4686.78–4714.48]	[1338.49–1378.29]	[2398.31–2430.46]	[7633.80–7684.83]
All‐cause hospitalizations within 28 days of first influenza diagnosis^a^ ^,^ ^b^				
Absolute number of hospitalizations (all cases)	27,587	1161	4713	21,713
Incidence proportion per 100,000 individuals	307.39	89.34	134.60	520.24
[95% CI]	[303.76–311.01]	[84.20–94.48]	[130.76–138.44]	[513.33–527.14]

Abbreviations: CI, confidence interval; ER, emergency room; HCRU, healthcare resource utilization; ICD‐10, International Classification of Disease–10^th^ revision; ICU, intensive care unit.

^a^ Defined as ICD‐10 diagnostic codes J09–J11.

^b^ If an individual had multiple influenza‐related ER visits or outpatient visits or was hospitalized multiple times for influenza or its potential complications within the influenza season, these were considered to be the same HCRU event.

^c^ Defined as ICD‐10 diagnostic codes J09–J18.

^d^ Defined as ICD‐10 diagnostic codes J09–J18, J40–J45, or J96.

^e^ Defined as ICD‐10 diagnostic codes G45, G46, I20, I21, I24, I25, I50, I60–I63, I65, I66, I69, I67.8, I67.9, J09–J11, J40–J45, or J96.

When HCRU was assessed by age group, the incidence proportion of influenza‐related ER visits increased with age, being highest in individuals aged ≥ 75 years, whereas the incidence proportion of influenza‐related outpatient visits was highest among individuals aged ≥ 60 to < 65 years (Table 2). The incidence proportion of hospitalizations for influenza per 100,000 per season also tended to increase with age, being highest in those aged ≥ 75 years. The same trend was observed for the incidence proportion of hospitalizations for influenza broadly defined, respiratory disease, respiratory or cardiovascular disease, and for all‐cause within 28 days of the first influenza diagnosis.

When assessed in each influenza season, the average HCRU for influenza or its potential complications generally increased over time (Table S6).

Among individuals aged ≥ 60 to < 65 years with ≥ 1 comorbidity, HCRU (across all four seasons) for influenza or its potential complications was higher than that observed in all individuals aged ≥ 60 to < 65 years and in those aged ≥ 60 to < 65 years without comorbidities (Table S7).

Most hospitalizations for influenza and its potential complications were of ≤ 60 days in duration (Figure S2).

### 3.3 Probability of Mortality

Among individuals with mortality data (*N* = 1,758,000), 3971 were hospitalized for influenza and 274 died within 30 days of initial hospitalization (Table 3). The probability of 30‐day inpatient mortality following hospitalization for influenza, influenza broadly defined, respiratory disease, and respiratory or cardiovascular disease was 6.90% (*n* = 274), 13.81% (*n* = 3638), 14.14% (*n* = 6550), and 9.02% (*n* = 9204), respectively. The probability of 30‐day inpatient mortality following all‐cause hospitalizations within 28 days of the first influenza diagnosis was 7.64% (*n* = 490).

**TABLE 3:** Probability of inpatient mortality within 30 days of hospitalization due to influenza or its potential complications.

	All individuals with mortality data *N* = 1,758,000	Age group, years		
		≥ 60 to < 65 *n* = 192,636	≥ 65 to < 75 *n* = 497,546	≥ 75 *n* = 1,067,818
Hospitalizations for influenza^a^ ^,^ ^b^	*n* = 3971	*n* = 76	*n* = 309	*n* = 3586
Absolute number of deaths	274	3	24	247
Probability of 30‐day inpatient mortality, %	6.90	3.95	7.77	6.89
[95% CI]	[6.11–7.69]	[0.00–8.33]	[4.78–10.75]	[6.06–7.72]
Hospitalizations for influenza broadly defined (influenza or pneumonia)^b^ ^,^ ^c^	*n* = 26,341	*n* = 463	*n* = 2292	*n* = 23,586
Absolute number of deaths	3638	51	319	3268
Probability of 30‐day inpatient mortality, %	13.81	11.02	13.92	13.86
[95% CI]	[13.39–14.23]	[8.16–13.87]	[12.50–15.34]	[13.41–14.30]
Hospitalizations for respiratory disease^b^ ^,^ ^d^	*n* = 46,336	*n* = 1023	*n* = 5003	*n* = 40,310
Absolute number of deaths	6550	108	718	5724
Probability of 30‐day inpatient mortality, %	14.14	10.56	14.35	14.20
[95% CI]	[13.82–14.45]	[8.67–12.44]	[13.38–15.32]	[13.86–14.54]
Hospitalizations for respiratory or cardiovascular disease^b^ ^,^ ^e^	*n* = 102,004	*n* = 2619	*n* = 11,986	*n* = 87,399
Absolute number of deaths	9204	153	1040	8011
Probability of 30‐day inpatient mortality, %	9.02	5.84	8.68	9.17
[95% CI]	[8.85–9.20]	[4.94–6.74]	[8.17–9.18]	[8.97–9.36]
All‐cause hospitalizations within 28 days of first influenza diagnosis^a^ ^,^ ^b^	*n* = 6414	*n* = 165	*n* = 626	*n* = 5623
Absolute number of deaths	490	5	46	439
Probability of 30‐day inpatient mortality, %	7.64	3.03	7.35	7.81
[95% CI]	[6.99–8.29]	[0.41–5.65]	[5.30–9.39]	[7.11–8.51]

Abbreviations: CI, confidence interval; HCRU, healthcare resource utilization; ICD‐10, International Classification of Disease–10^th^ revision.

^a^ Defined as ICD‐10 diagnostic codes J09–J11.

^b^ If an individual had multiple influenza‐related ER visits or outpatient visits or was hospitalized multiple times for influenza or its potential complications within the influenza season, these were considered to be the same HCRU event.

^c^ Defined as ICD‐10 diagnostic codes J09–J18.

^d^ Defined as ICD‐10 diagnostic codes J09–J18, J40–J45, or J96.

^e^ Defined as ICD‐10 diagnostic codes G45, G46, I20, I21, I24, I25, I50, I60–I63, I65, I66, I69, I67.8, I67.9, J09–J11, J40–J45, or J96.

The probability of 30‐day inpatient mortality following hospitalization for influenza, influenza broadly defined, respiratory disease, and respiratory or cardiovascular disease, as well as following all‐cause hospitalizations within 28 days of the first influenza diagnosis, was higher among individuals aged ≥ 65 to < 75 years or ≥ 75 years than in those aged ≥ 60 to < 65 years (Table 3).

In sensitivity analyses, the same trends were observed when the probability of inpatient mortality was assessed within 60 or 90 days of admission (Tables S8 and S9, respectively).

The probability of 30‐day inpatient mortality in each individual influenza season and the probability of 30‐, 60‐, and 90‐day inpatient mortality among all individuals aged ≥ 60 to < 65 years and in those aged ≥ 60 to < 65 years with ≥ 1 comorbidity are shown in Tables S10 and S11, respectively.

**FIGURE 2:**
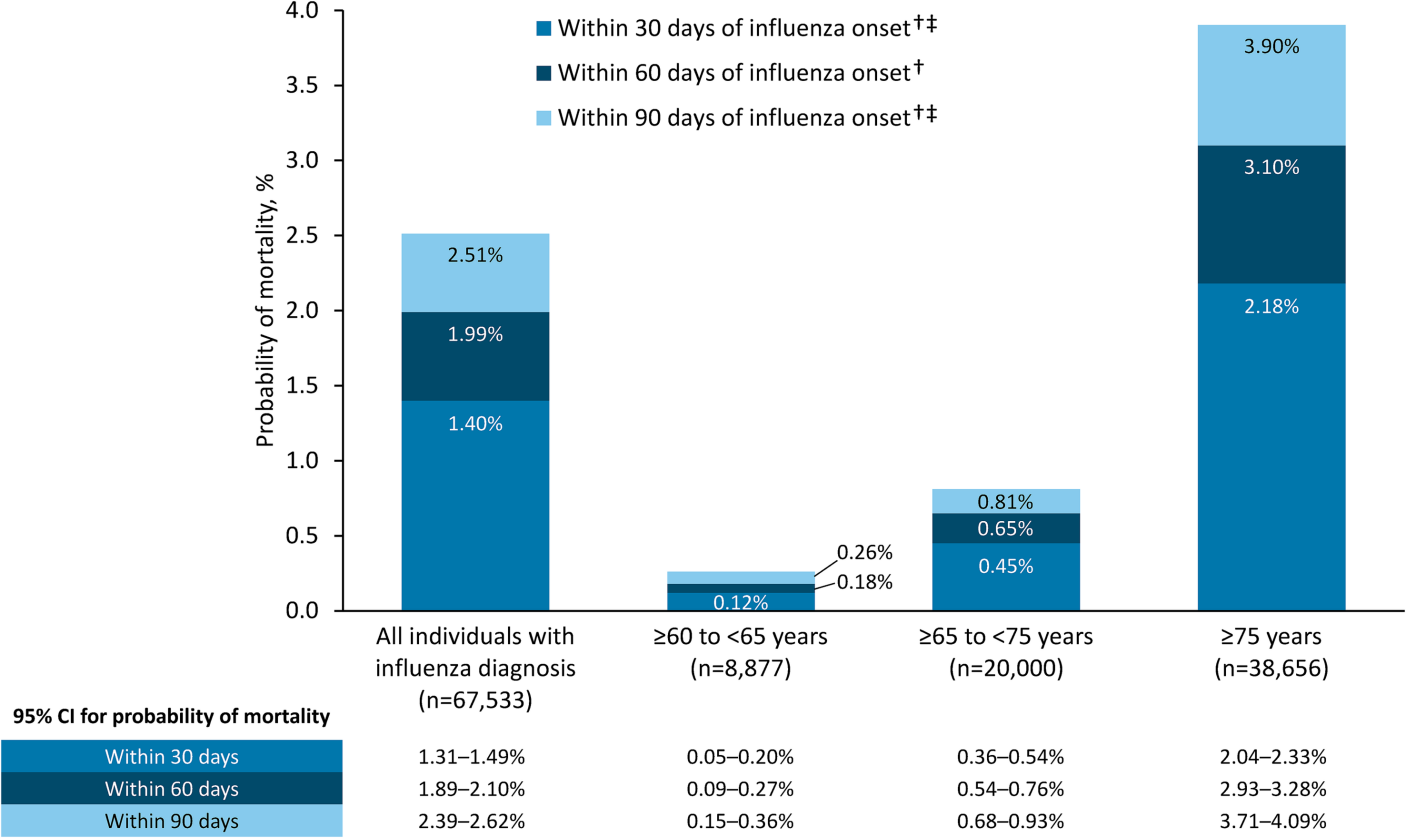
Probability of mortality within 30, 60, or 90 days of influenza onset among individuals with mortality data and influenza diagnosis. †Defined as International Classification of Disease–10^th^ revision diagnostic codes J09–J11. ‡Sensitivity analysis. CI, confidence interval.

Among individuals with mortality data who were diagnosed with influenza (*N* = 67,533), 1.40% of individuals died within 30 days of influenza onset (Figure 2). The probability of influenza‐related mortality within 30 days was highest in individuals aged ≥ 75 years; sensitivity analyses showed a similar trend for the probability of 60‐ and 90‐day influenza‐related mortality (Figure 2).

### 3.4 Direct Medical Costs

The mean ± SD and median (Q1–Q3) direct medical costs per HCRU event associated with influenza or its potential complications are summarized in Tables 4 and S12, respectively. The mean ± SD total costs per individual for ER (¥40,975.1 ± 17,190.3) and outpatient (¥13,594.0 ± 11,746.5) visits for influenza were lower than the 60‐day costs per individual for hospitalizations for influenza (¥679,552.4 ± 902,915.1). The mean ± SD 60‐day medical costs per individual for influenza potential complications ranged from ¥795,620.2 ± 893,810.6 to ¥898,716.6 ± 990,689.9.

**TABLE 4:** Mean direct medical costs associated with healthcare resource utilization for influenza or its potential complications.

Mean ± SD medical costs, ¥	Total study population^a^ *N* = 8,974,708	Age group, years		
		≥ 60 to < 65 *n* = 1,299,552	≥ 65 to < 75 *n* = 3,501,469	≥ 75 *n* = 4,173,687
Total costs per event				
ER visit for influenza^b^ ^,^ ^c^	40,975.1 ± 17,190.3	38,429.4 ± 16,175.1	40,640.8 ± 16,335.2	41,160.9 ± 17,385.6
Outpatient visit for influenza^b^ ^,^ ^c^	13,594.0 ± 11,746.5	12,226.7 ± 12,270.7	12,658.0 ± 10,197.9	15,040.9 ± 12,684.0
60‐day costs per hospitalization				
Hospitalization for influenza^b^ ^,^ ^c^	679,552.4 ± 902,915.1	817,528.1 ± 1,146,961.6	823,410.9 ± 1,626,554.0	649,549.6 ± 689,631.4
Hospitalization for influenza broadly defined (influenza or pneumonia)^c^ ^,^ ^d^	795,620.2 ± 893,810.6	945,445.1 ± 1,357,674.7	905,110.9 ± 1,237,171.7	770,369.7 ± 790,632.7
Hospitalization for respiratory disease^c^ ^,^ ^e^	859,025.4 ± 960,972.6	1,016,978.0 ± 1,294,437.8	995,631.6 ± 1,280,106.3	821,204.6 ± 849,723.3
Hospitalization for respiratory or cardiovascular disease^c^ ^,^ ^f^	898,716.6 ± 990,689.9	989,030.9 ± 1,222,902.5	996,119.6 ± 1,239,460.6	867,975.4 ± 895,820.6
All‐cause hospitalization within 28 days of first influenza diagnosis^b^ ^,^ ^c^	715,812.2 ± 888,657.8	779,163.4 ± 1,157,676.8	814,030.2 ± 1,359,267.3	691,108.6 ± 726,562.6

Abbreviations: ER, emergency room; HCRU, healthcare resource utilization; ICD‐10, International Classification of Disease–10^th^ revision; ICU, intensive care unit; SD, standard deviation; ¥, Yen.

^a^ Individuals with unknown or no medical costs were excluded from this analysis.

^b^ Defined as ICD‐10 diagnostic codes J09–J11.

^c^ If an individual had multiple influenza‐related ER visits or outpatient visits or was hospitalized multiple times for influenza or its potential complications within the influenza season, these were considered to be the same HCRU event.

^d^ Defined as ICD‐10 diagnostic codes J09–J18.

^e^ Defined as ICD‐10 diagnostic codes J09–J18, J40–J45, or J96.

^f^ Defined as ICD‐10 diagnostic codes G45, G46, I20, I21, I24, I25, I50, I60–I63, I65, I66, I69, I67.8, I67.9, J09–J11, J40–J45, or J96.

The total medical costs per ER or outpatient visit for influenza were slightly higher in individuals aged ≥ 75 years than in younger individuals (Table 4). However, the 60‐day medical costs per hospitalization for influenza, influenza broadly defined, respiratory disease, or respiratory or cardiovascular disease and all‐cause hospitalizations within 28 days of the first influenza diagnosis were slightly lower in individuals aged ≥ 75 years than in younger individuals.

In sensitivity analyses, the 30‐ and 90‐day medical costs associated with hospitalizations for influenza and its potential complications were also slightly lower in the ≥ 75 years age group than in younger patients (Tables S13 and S14, respectively).

The medical costs for each influenza season and in individuals aged ≥ 60 to < 65 years with ≥ 1 comorbidity are shown in Tables S15 and S16, respectively.

## 4 Discussion

This large retrospective database study describes the overall trends in seasonal influenza between 2015 and 2019 among Japanese individuals aged ≥ 60 years, highlighting the burden of disease with regard to HCRU, mortality, and costs.

Across the four influenza seasons, HCRU for influenza was high, with incidence proportions per 100,000 individuals per season of 17.18 for ER visits, 3893.53 for outpatient visits, and 181.50 for hospitalizations. The burden of hospitalizations for the potential complications of influenza was also substantial, with incidence proportions per 100,000 individuals per season of 1242.50 for influenza broadly defined, 2176.49 for respiratory disease, and 4700.63 for respiratory or cardiovascular disease. The incidence proportion of all‐cause hospitalizations within 28 days of the first influenza diagnosis was also high (307.39 per 100,000 individuals per season). When HCRU was examined by age, individuals aged ≥ 60 to < 65 years had a higher incidence proportion of outpatient visits for influenza than those aged ≥ 65 to < 75 or ≥ 75 years, whereas individuals aged ≥ 75 years had the highest incidence proportion of ER visits and hospitalizations for influenza or its potential complications and all‐cause hospitalizations within 28 days of the first influenza diagnosis. Similarly, a previous Japanese study by Hagiwara and colleagues reported a lower proportion of outpatient visits (including ER visits) for clinically diagnosed influenza in individuals aged ≥ 85 years (22.7%) than in those aged 65–74 years (31.4%) or 75–84 years (33.7%) but relatively more hospitalizations (36.1% vs. 22.2% and 35.5%, respectively) [26]. Our findings were also consistent with those of a systematic review by Taniguchi and colleagues, which reported that rates of laboratory‐confirmed influenza hospitalization increased with age among individuals aged 60–69 years (1300–2000 cases per season) or ≥ 70 years (8800–11,500 cases per season), with mortality rates being highest in older individuals [21].

Among individuals with mortality data, the probability of 30‐day inpatient mortality was highest among those who were hospitalized for respiratory disease (14.14%). In the age subgroup analysis, the 30‐day inpatient mortality probability was higher in individuals aged ≥ 65 to < 75 years or ≥ 75 years than in those aged ≥ 60 to < 65 years. These results were similar to those of the study by Hagiwara and colleagues, in which the probability of inpatient death was significantly higher among individuals with clinically diagnosed influenza who were aged ≥ 85 years than in those aged 60–64 years (18.6% vs. 9.9%; *p* < 0.001) [26].

The direct medical costs associated with hospitalization for influenza and its potential complications were assessed 60 days after admission. This interval was chosen because most hospitalizations had a duration of ≤ 60 days. In addition, if two or more hospitalizations occurred in the same individual within the influenza season, these were considered as a single event. The 60‐day medical costs associated with hospitalization for influenza were higher than the total medical costs for ER and outpatient visits for influenza. Across the influenza seasons, the range of mean hospitalization costs per individual was ¥821,403.0–¥899,935.2 for respiratory disease and ¥870,582.6–¥922,814.9 for respiratory or cardiovascular disease. These costs are comparable with those reported in previous European studies [27, 28, 29]. A French study reported that the total excess cost of hospitalization for respiratory disease attributed to influenza in the 2017–2018 season was €217.4 million among 40,045 individuals (i.e., cost per individual €5428.9) [27]. Assuming a currency conversion factor of €1 = ¥174.4, this corresponds to a per‐individual cost of approximately ¥946,800. In an Italian study, the per individual cost of hospitalization for cardiorespiratory events related to influenza was €4035.32 (corresponding to ¥703,759.8) [28], whereas a study from the Netherlands indicated that the hospitalization cost per cardiorespiratory episode was €5629.72 (corresponding to ¥981,823.2) [29]. In the age subgroups, the medical costs of ER and outpatient visits for influenza were slightly higher in individuals aged ≥ 75 years than in younger individuals, possibly because of the higher prevalence of ≥ 1 comorbidity in the older age group (62.4% vs. 33.9% and 44.2%, respectively). In contrast, the 60‐day costs of hospitalization for influenza or its potential complications and all‐cause hospitalizations within 28 days of the first influenza diagnosis were slightly lower in individuals aged ≥ 75 years than in those aged ≥ 60 to < 65 or ≥ 65 to < 75 years. Of note, this cost analysis found large SD values, indicating that the data points have a wide distribution relative to the mean value. This is a typical trend for cost data, which normally have a right‐skewed distribution; similar findings were reported in a previous HRCU cost analysis of respiratory syncytial virus in Japanese children [30].

When assessed by influenza season, the HCRU for influenza or its potential complications generally increased over time, whereas the probability of inpatient mortality and medical costs remained similar. The increase in influenza burden over seasons is consistent with official Japanese surveillance data [31, 32], and it would be interesting to further confirm this trend for seasons following the COVID‐19 pandemic.

Underlying chronic comorbidities are known to be associated with an increased risk of influenza potential complications [9, 14], potentially leading to increased annual HCRU and associated costs [33]. Among individuals aged ≥ 60 to < 65 years with ≥ 1 comorbidity, HCRU and the probability of 30‐day inpatient mortality due to influenza and its potential complications were higher than in all individuals aged ≥ 60 to < 65 years or individuals aged ≥ 60 to < 65 years without comorbidities. However, the 60‐day medical costs per individual for hospitalizations due to influenza or its potential complications were generally comparable between individuals aged ≥ 60 to < 65 years with ≥ 1 comorbidity and all individuals aged ≥ 60 to < 65 years.

The substantial burden of influenza among Japanese individuals aged ≥ 60 years highlights the need for more effective prevention measures in this population, including the implementation of vaccination programs with enhanced influenza vaccines. Among these, high‐dose inactivated influenza vaccine (IIV‐HD), which contains four times more antigen than standard‐dose inactivated influenza vaccine (IIV‐SD), is the only vaccine to have demonstrated improved efficacy against influenza cases compared with IIV‐SD in older individuals (aged ≥ 65 years), as well as lower rates of hospitalization for influenza potential complications in randomized controlled trial settings [34, 35, 36]. As of August 2022, quadrivalent IIV‐HD (IIV4‐HD) was approved in 33 countries worldwide [20]. There is also a need to increase vaccination coverage rates. Of note, the influenza vaccination rates per season for individuals aged ≥ 60 years in Japan for the duration of this study ranged from 47.9% to 50.9% [37]. Increased awareness of the considerable burden of influenza among older individuals is a key step toward achieving more effective prevention measures, and successful IIV‐HD vaccination programs are expected to reduce severe outcomes and HCRU during the influenza season in Japan, as has been observed in other countries [36].

The main strength of this study is the inclusion of data from almost 9 million individuals aged ≥ 60 years over four influenza seasons, allowing for assessment of the broad burden of influenza in this population. However, the following study limitations also need to be considered. Data from the three insurance associations were not linked to each other in the DeSC database, so the same insured individual may have been counted more than once across different seasons, possibly leading to inaccurate estimation of HCRU (e.g., in individuals with an extremely high risk of hospitalization). However, overlap within the same influenza season was not expected, because individuals are not enrolled in multiple health insurance plans simultaneously under the Japanese health insurance system and because the study inclusion criteria only used data from the health insurance plan in which the individual was enrolled during the entire baseline period. In addition, there is limited evidence supporting the generalizability of the DeSC database to the wider Japanese population. However, it has been shown that the distribution of age and sex in the DeSC data is comparable with that of the Japanese population and that its representativeness is considered to be superior to databases derived from other sole insurers [25, 38]. Furthermore, similar to other database studies, medical coding may not always reflect true diagnoses or procedures. Of note, mortality data were unavailable for > 80% of the study population as this information has only recently been provided by insurers to the DeSC database and insurance claims data do not track all out‐of‐hospital deaths. Moreover, according to the protocol, if multiple HCRU events occurred within an influenza season, they were counted as a single event, meaning that the burden of influenza and its potential complications may have been underestimated. Finally, data on vaccination status were not available, which may have influenced our results; nevertheless, our data reflect the real‐world disease burden of influenza with vaccination rates during the study period (i.e., 2015–2019; 49.3%) [37].

## 5 Conclusions

In this real‐world database study of older Japanese individuals (aged ≥ 60 years), seasonal influenza and its potential complications represented a substantial burden with regard to HRCU, mortality, and medical costs that increased with age. These findings emphasize the need for better strategies for the prevention of seasonal influenza in older adults in Japan.

## Author Contributions

**Takeshi Arashiro:** methodology, formal analysis, data curation, validation, writing – original draft, supervision, writing – review and editing. **Yuki Tajima:** methodology, formal analysis, writing – review and editing. **Yohei Ban:** software, data curation, writing – review and editing. **Matthew Loiacono M:** formal analysis, methodology, writing – original draft, writing – review and editing. **Masayo Ideguchi:** formal analysis, validation, writing – original draft, writing – review and editing, project administration. **Caroline de Courville:** methodology, formal analysis, validation, supervision, visualization, project administration, writing – review and editing, writing – original draft.

## Disclosure

All named authors meet the International Committee of Medical Journal Editors (ICMJE) criteria for authorship, take responsibility for the integrity of the work as a whole, and have given their approval for this version to be published.

## Ethics Statement

This noninterventional study was conducted in accordance with the Ethical Guidelines for Research in the Life Sciences and Medical Sciences on Human Subjects. The ethics committee of SOUKEN Co., Ltd. reviewed and approved the study prior to study initiation.

## Consent

The study used deidentified data from an insurance claims database; therefore, the requirement for informed consent was waived because of the anonymous nature of the database.

## Conflicts of Interest

Takeshi Arashiro is a cooperative researcher with the National Institute of Infectious Diseases, Japan, and is an employee of Sanofi K.K. Yuki Tajima is an employee of Sanofi K.K. Yohei Ban has received consultancy fees from Sanofi K.K. and is an employee of Intage Healthcare Inc. Masayo Ideguchi is a full‐time employee of Sanofi K.K. and holds stock and/or stock options in the company. Matthew M Loiacono and Caroline de Courville are full‐time employees of Sanofi and hold stock and/or stock options in the company.

## Supporting information


**Table S1.** Estimated number of insured individuals aged ≥60 years in the DeSC dataset versus the total Japanese population overall and by influenza season.
**Table S2.** Anti‐influenza drugs.
**Table S3.** Definitions of outcomes.
**Table S4.** Baseline characteristics of individuals with mortality data.
**Table S5.** Baseline characteristics of individuals with influenza.
**Table S6.** Average healthcare resource utilization events per season for influenza or its potential complications in each influenza season.
**Table S7.** Average healthcare resource utilization events per season (across all four seasons) for influenza or its potential complications in all individuals aged ≥60 to <65 years and in those with ≥1 comorbidity or without comorbidities.
**Table S8.** Probability of inpatient mortality within 60 days of hospitalization for influenza or its potential complications (sensitivity analysis).
**Table S9.** Probability of inpatient mortality within 90 days of hospitalization for influenza or its potential complications (sensitivity analysis).
**Table S10.** Probability of inpatient mortality within 30 days of hospitalization due to influenza or its potential complications in each influenza season among individuals with available mortality data.
**Table S11.** Probability of inpatient mortality following hospitalization due to influenza or its potential complications in all individuals aged ≥60 to <65 years and in those with or without ≥1 comorbidity among individuals with available mortality data.
**Table S12.** Median direct medical costs associated with healthcare resource utilization for influenza or its potential complications.
**Table S13.** Mean 30‐day direct medical costs associated with hospitalization for influenza or its potential complications (sensitivity analysis).
**Table S14.** Mean 90‐day direct medical costs associated with hospitalization for influenza or its potential complications (sensitivity analysis).
**Table S15.** Mean direct medical costs associated with healthcare resource utilization for influenza or its potential complications in each individual influenza season.
**Table S16.** Direct medical costs associated with healthcare resource utilization for influenza or its potential complications in all individuals aged ≥60 to <65 years and in those with ≥1 comorbidity or without comorbidities.
**Figure S1.** Definitions of the four influenza seasons and associated baseline periods (i.e., before the start of each influenza season) during the study period.
**Figure S2.** Distribution of the duration for hospitalizations for (a) influenza†‡ (*N* = 16,289), (b) influenza broadly defined (influenza or pneumonia)‡§ (*N* = 111,511), (c) respiratory disease‡¶ (*N* = 195,334), and (d) respiratory or cardiovascular disease‡# (*N* = 421,868) across all four seasons in the total study population.

## Data Availability

The data that support the findings of this study are available from DeSC Healthcare, Inc. (Tokyo, Japan). Restrictions apply to the availability of these data, which were used under license for this study. Data are available from the corresponding author with the permission of DeSC Healthcare, Inc.
