# Supplementary material for: The Burden of Seasonal Influenza and Its Potential Complications Among Older Japanese Adults: A Real‐World Database Study
Source: Influenza Other Respir Viruses. 2024 Nov 12;18(11):e70032. doi: 10.1111/irv.70032 (PMC11557991; doi:10.1111/irv.70032)
Supplement: Supplementary file 1 — Table S1. Estimated number of insured individuals aged ≥60 years in the DeSC dataset versus the total Japanese population overall and by influenza season. Table S2. Anti‐influenza drugs. Table S3. Definitions of outcomes. Table S4. Baseline characteristics of individuals with mortality data. Table S5. Baseline characteristics of individuals with influenza. Table S6. Average healthcare resource utilization events per season for influenza or its potential complications in each influenza season. Table S7. Average healthcare resource utilization events per season (across all four seasons) for influenza or its potential complications in all individuals aged ≥60 to <65 years and in those with ≥1 comorbidity or without comorbidities. Table S8. Probability of inpatient mortality within 60 days of hospitalization for influenza or its potential complications (sensitivity analysis). Table S9. Probability of inpatient mortality within 90 days of hospitalization for influenza or its potential complications (sensitivity analysis). Table S10. Probability of inpatient mortality within 30 days of hospitalization due to influenza or its potential complications in each influenza season among individuals with available mortality data. Table S11. Probability of inpatient mortality following hospitalization due to influenza or its potential complications in all individuals aged ≥60 to <65 years and in those with or without ≥1 comorbidity among individuals with available mortality data. Table S12. Median direct medical costs associated with healthcare resource utilization for influenza or its potential complications. Table S13. Mean 30‐day direct medical costs associated with hospitalization for influenza or its potential complications (sensitivity analysis). Table S14. Mean 90‐day direct medical costs associated with hospitalization for influenza or its potential complications (sensitivity analysis). Table S15. Mean direct medical costs associated with healthcare resource utilization for [file IRV-18-e70032-s001.docx]

# Supporting information

## Supplementary methods

### Insurance associations in the DeSC database

Kempo (employer-based health insurance association) can be divided in three schemes. The first of these is managed by Health Insurance Societies, aimed mainly employees of large companies, provided by >1,300 insurers, and eligible for public subsidies in individuals with financial difficulties. The second scheme is managed by Mutual Aid Associations and aimed at government workers, but is ineligible for public subsidies. The third scheme is administered by the Japan Health Insurance Association and aimed at employees of small- to medium-sized companies.

Kokuho (residence-based National Health Insurance [NHI] association) provides health insurance for self-employed or unemployed individuals, and retirees aged <75 years. The NHI insures individuals who are not otherwise enrolled in insurance schemes.

Koki Koreisha Iryo Seido (the Advanced Elderly Medical Service System) insures all people aged ≥75 years, and all enrollees become insured individuals, with no distinction between supporters and dependents.

### Definition of influenza potential complications

Influenza potential complications were the same as those defined in a previous randomized controlled study that assessed the relative vaccine efficacy of high-dose inactivated influenza vaccine versus standard dose.^1^ Among all serious adverse events (SAEs) observed in this previous study, cardiorespiratory SAEs considered as potentially related to influenza were identified by two blinded physicians. The original Medical Dictionary for Regulatory Activities (MedDRA) preferred terms for these cardiorespiratory SAEs were converted to International Classification of Disease – 10^th^ revision (ICD) diagnostic codes, which were used to identify individuals with influenza or its potential complications in this study (Supplementary Table S2).

### Calculation of endpoints

For the primary endpoints (1) to (3), the average incidence proportion per 100,000 individuals per season (across all four seasons) was calculated using the following equation:

$$Incidence proportion per 100,000 individuals per season=\left( \frac{no. of individuals with event}{total no. of individuals} \right)\times100,000$$

For primary endpoint (4), the probability of 30-day inpatient mortality following hospitalization for influenza or its potential complications (i.e., influenza, influenza broadly defined, respiratory disease, or respiratory or cardiovascular disease) was calculated as a percentage using the following equation:

$$Probability of 30-day inpatient mortality=\left( \frac{no. of inpatient deaths within 30 days of hospitalization}{total no. of individuals hospitalized} \right)\times100\%$$

For the secondary endpoints, the overall probability of mortality among all individuals with influenza within 30 days of symptom onset was calculated as a percentage using the following equation:

$$Probability of 30-day influenza-related mortality=\left( \frac{no. of deaths within 30 days of influenza onset}{no. of individuals with influenza and mortality data} \right)\times100\%$$

## Supplementary Table S1.

Estimated number of insured individuals aged ≥60 years in the DeSC dataset versus the total Japanese population overall and by influenza season

|  | **Overall 2015–2019** | **Influenza season** | | | |
| --- | --- | --- | --- | --- | --- |
|  |  | **2015/2016** | **2016/2017** | **2017/2018** | **2018/2019** |
| Insured individuals aged ≥60 years enrolled in the DeSC database,† n | 9,725,834 | 941,912 | 2,153,040 | 2,469,513 | 4,161,369 |
| Individuals aged ≥60 years in the Japanese population (× 1,000),‡ N | 171,297 | 42,421 | 42,753 | 42,955 | 43,168 |
| Proportion of individuals in Japanese population enrolled in the DeSC database, % | 5.68 | 2.22 | 5.04 | 5.75 | 9.64 |

†At start of each influenza season; ‡Data from Statistics Bureau of Japan Statistics Bureau (<https://www.stat.go.jp/english/index.html>)

## Supplementary Table S2.

Anti-influenza drugs

| **Drug name** | **ATC classification code** |
| --- | --- |
| Oseltamivir (Tamiflu®) |  |
| Capsules 75 mg | 610443074 |
| Dry syrup 3% | 610462002 |
| Oseltamivir [Sawai] |  |
| Capsules 75 mg | 622638801 |
| Dry syrup 3% | 622638901 |
| Baloxavir marboxil (Xofluza®) |  |
| Tablets 10 mg | 622622501 |
| Tablets 20 mg | 622622601 |
| Peramivir (Rapiacta®) |  |
| For IV drip infusion 300 mg/60 mL bag | 621972102 |
| For IV drip infusion 150 mg/15 mL vial | 621972202 |
| Zanamivir (Relenza®) |  |
| For oral inhalation 5 mg | 660443018 |
| Laninamivir (Inavir®) |  |
| Dry powder inhaler 20 mg | 622012101 |
| For inhalation suspension set 160 mg | 622688601 |

ATC, Anatomical Therapeutic Chemical; IV, intravenous

## Supplementary Table S3.

Definitions of outcomes

| **Outcome** | **Definition** |
| --- | --- |
| **Influenza** | ICD-10 diagnostic codes: J09–J11 (‘Influenza viral infections’)† or an anti-influenza drug is prescribed during the influenza season‡  Diagnosis date was one of the following: date of anti-influenza drug prescription, start date of medical treatment for influenza, or first day of month of influenza diagnosis (if there is no prescription date or treatment start date) |
| **Influenza potential complications** |  |
| Influenza broadly defined (influenza or pneumonia) | ICD-10 diagnostic codes: J09–J11 (‘Influenza viral infections’)† and J12–J18 (‘Lower respiratory tract and lung infections’)† |
| Respiratory disease | ICD-10 diagnostic codes: J09–J11 (‘Influenza viral infections’),† J12–J18 (‘Lower respiratory tract and lung infections’),† J40–J45 (‘Bronchospasm and obstruction’),† J96 (‘Respiratory failure [excluding neonatal]’)† |
| Respiratory and cardiovascular disease | ICD-10 diagnostic codes: G45, G46 (‘Transient cerebrovascular events’),† I20 (‘Ischemic coronary artery disorders’),† I21, I24, I25 (‘Coronary artery disorders NEC’),† I50 (‘Heart failures NEC’),† I60–I63, I65, I66, I67.8, I67.9, I69 (‘Central nervous system hemorrhages and cerebrovascular accidents’),† J09–J11 (‘Influenza viral infections’)†, J40–J45 (‘Bronchospasm and obstruction’),† or J96 (‘Respiratory failure [excluding neonatal]’)† |
| **HCRU for influenza** |  |
| ER visits | The disease/injury (i.e., ICD-10 diagnostic code) on the medical fee statement for the ER visit corresponded to “influenza”  The medical fee statement included the date of prescription of anti-influenza medications, the start date of medical treatment for influenza (not suspected), and the date of any influenza test§  If an individual had multiple influenza-related ER visits within the influenza season, it was considered to be the same ER visit |
| Outpatient visits | The disease/injury (i.e., ICD-10 diagnostic code) on the medical fee statement for the outpatient visit corresponded to “influenza” (excluded emergency room visits)  The medical fee statement included the date of prescription of anti-influenza medications, the start date of medical treatment for influenza (not suspected), and the date of any influenza test§  If an individual had multiple influenza-related outpatient visits within the influenza season it was considered to be the same outpatient visit |
| Hospitalizations | The disease/injury (i.e., ICD-10 diagnostic code) in the medical fee statement for hospitalization corresponded to “influenza” (cases where the admission date was earlier than the influenza onset date were excluded)  If an individual was hospitalized for influenza or its potential complications multiple times within the influenza season, it was considered to be the same hospitalization |
| **Hospitalization for influenza potential complications** | |
| Influenza broadly defined (influenza or pneumonia) | The disease/injury (i.e., ICD-10 diagnostic code) on the medical fee statement for hospitalization corresponded to “influenza broadly defined (influenza or pneumonia)” |
| Respiratory disease | The disease/injury (i.e., ICD-10 diagnostic code) on the medical fee statement for hospitalization corresponded to “respiratory disease” |
| Respiratory and cardiovascular disease | The disease/injury on the medical fee statement for hospitalization corresponded to “respiratory and cardiovascular disease” |
| **All-cause hospitalizations within 28 days of initial influenza diagnosis** | |
| All cases | Any hospitalization occurring within 28 days after the date of influenza onset was defined as having the date of admission within the period of influenza onset plus 27 days (regardless of disease/injury on medical fee statement) |
| **Mortality** |  |
| Deaths of hospitalized individuals due to influenza or its potential complications | Deaths of individuals who were hospitalized due to influenza or its potential complications that occurred within 59 days of admission |
| Deaths due to influenza | Deaths that occurred within 59 days of the first influenza onset (or if first influenza onset was in the same month and year as the end of the observable period) |
| **Direct medical costs** |  |
| Medical costs of hospitalization for influenza or its potential complications per individual | Sum of medical practice and pharmaceutical costs per individual, as reported on the medical fee statement for all hospitalizations up to 59 days from admission for the first hospitalization for influenza or its potential complications¶ |
| Medical costs of outpatient visit for influenza per individual | Sum of medical practice and pharmaceutical costs per individual, as reported on the medical fee statement for all “outpatient visits” due to influenza during the influenza season# |
| Medical costs of ER visit for influenza per individual | Sum of medical practice and pharmaceutical costs per individual, as reported on the medical fee statement for “ER visits” due to influenza during the influenza season |

†MedDRA PT equivalent, as defined by DiazGranados et al., 2015^1^ (see Supplementary methods); ‡If an individual had multiple influenza-related ER visits or outpatient visits or was hospitalized multiple times for influenza or its potential complication within the influenza season, these were considered to be the same HCRU event; §Included viral antibody titers for influenza virus types A or B (qualitative, semi-quantitative, or quantitative), influenza virus antigen (qualitative), influenza nucleic acid detection, and SARS-CoV-2 test with simultaneous detection of influenza nucleic acid; ¶If the date of hospital discharge is on or after the admission date + 59 days, medical costs for the admission date + 59 days were calculated; #Costs were calculated from total medical costs of outpatient visits for influenza (including total general practitioner costs)

ER, emergency room; HCRU, healthcare resource utilization; ICD-10, International Classification of Disease – 10^th^ revision; ICU, intensive care unit; MedDRA, Medical Dictionary for Regulatory Activities; PT, preferred term; SARS-CoV-2, severe acute respiratory syndrome coronavirus 2

## Supplementary Table S4.

Baseline characteristics of individuals with mortality data

|  | **Individuals with mortality data**  **N=1,758,000** | **Age group, years** | | |
| --- | --- | --- | --- | --- |
|  |  | **≥60 to <65**  **n=192,636** | **≥65 to <75**  **n=497,546** | **≥75**  **n=1,067,818** |
| Male sex, n (%) | 743,415 (42.3) | 85,809 (44.5) | 235,340 (47.3) | 422,266 (39.5) |
| Age, mean ± SD, years | 75.8 ± 8.9 | – | – | – |
| Type of insurance, n (%) |  |  |  |  |
| Kempo† | 678,010 (38.6) | 192,636 (100.0) | 485,374 (97.6) | 0 |
| Koki Koreisha Iryo Seido‡ | 1,079,990 (61.4) | 0 | 12,172 (2.4) | 1,067,818 (100.0) |
| Comorbidities, n (%) |  |  |  |  |
| ≥1 comorbidity | 973,235 (55.4) | 67,323 (35.0) | 222,767 (44.8) | 683,145 (64.0) |
| Diabetes mellitus | 512,405 (29.1) | 35,910 (18.6) | 127,297 (25.6) | 349,198 (32.7) |
| Cancer | 323,393 (18.4) | 20,783 (10.8) | 71,539 (14.4) | 231,071 (21.6) |
| Ischemic heart disease | 283,118 (16.1) | 10,351 (5.4) | 44,926 (9.0) | 227,841 (21.3) |
| Asthma | 179,852 (10.2) | 12,299 (6.4) | 37,443 (7.5) | 130,110 (12.2) |
| Atrial fibrillation | 110,646 (6.3) | 3,217 (1.7) | 15,880 (3.2) | 91,549 (8.6) |
| Congestive heart failure | 98,875 (5.6) | 2,837 (1.5) | 11,107 (2.2) | 84,931 (8.0) |
| Chronic renal failure | 78,054 (4.4) | 3,209 (1.7) | 12,325 (2.5) | 62,520 (5.9) |
| COPD | 60,474 (3.4) | 1,731 (0.9) | 9,191 (1.8) | 49,552 (4.6) |
| Pneumonia | 51,231 (2.9) | 2,111 (1.1) | 7,499 (1.5) | 41,621 (3.9) |
| Parkinson’s disease | 38,417 (2.2) | 3,797 (2.0) | 7,430 (1.5) | 27,190 (2.5) |
| Acute respiratory failure | 11,060 (0.6) | 256 (0.1) | 1,249 (0.3) | 9,555 (0.9) |
| Acute renal failure | 3,839 (0.2) | 147 (0.1) | 509 (0.1) | 3,183 (0.3) |
| HIV/AIDS | 395 (0.02) | 58 (0.03) | 122 (0.02) | 215 (0.02) |

†The Health Insurance Association for employees of large companies; ‡The Advanced Elderly Medical Service System for individuals aged ≥75 years

AIDS, autoimmune deficiency syndrome; COPD, chronic obstructive pulmonary disease; HIV, human immunodeficiency virus; SD, standard deviation

## Supplementary Table S5.

Baseline characteristics of individuals with influenza

|  | **Individuals with influenza**  **N=370,430** | **Age group, years** | | |
| --- | --- | --- | --- | --- |
|  |  | **≥60 to <65**  **n=62,224** | **≥65 to <75**  **n=141,575** | **≥75**  **n=166,631** |
| Male sex, n (%) | 150,271 (40.6) | 23,156 (37.2) | 60,465 (42.7) | 66,650 (40.0) |
| Age, mean ± SD, years | 73.6 ± 9.3 | – | – | – |
| Type of insurance, n (%) |  |  |  |  |
| Kempo† | 11,942 (3.2) | 6,573 (10.6) | 5,369 (3.8) | 0 |
| Kokuho‡ | 185,479 (50.1) | 55,651 (89.4) | 129,828 (91.7) | 0 |
| Koki Koreisha Iryo Seido§ | 173,009 (46.7) | 0 | 6,378 (4.5) | 166,631 (100.0) |
| Comorbidities, n (%) |  |  |  |  |
| ≥1 comorbidity | 207,859 (56.1) | 24,417 (39.2) | 70,378 (49.7) | 113,064 (67.9) |
| Diabetes mellitus | 108,538 (29.3) | 12,364 (19.9) | 38,410 (27.1) | 57,764 (34.7) |
| Cancer | 62,493 (16.9) | 7,248 (11.7) | 22,073 (15.6) | 33,172 (19.9) |
| Ischemic heart disease | 59,584 (16.1) | 4,235 (6.8) | 16,103 (11.4) | 39,246 (23.6) |
| Asthma | 45,484 (12.3) | 6,345 (10.2) | 15,836 (11.2) | 23,303 (14.0) |
| Atrial fibrillation | 21,177 (5.7) | 1,057 (1.7) | 4,952 (3.5) | 15,168 (9.1) |
| Congestive heart failure | 18,564 (5.0) | 881 (1.4) | 3,322 (2.3) | 14,361 (8.6) |
| Chronic renal failure | 15,778 (4.3) | 1,098 (1.8) | 3,908 (2.8) | 10,772 (6.5) |
| Pneumonia | 13,184 (3.6) | 984 (1.6) | 2,897 (2.0) | 9,303 (5.6) |
| COPD | 11,563 (3.1) | 673 (1.1) | 3,027 (2.1) | 7,863 (4.7) |
| Parkinson’s disease | 8,015 (2.2) | 745 (1.2) | 1,734 (1.2) | 5,536 (3.3) |
| Acute respiratory failure | 3,307 (0.9) | 183 (0.3) | 664 (0.5) | 2,460 (1.5) |
| Acute renal failure | 757 (0.2) | 33 (0.05) | 170 (0.1) | 554 (0.3) |
| HIV/AIDS | 72 (0.02) | 22 (0.04) | 21 (0.01) | 29 (0.02) |

†The Health Insurance Association for employees of large companies; ‡The National Health Insurance Association for self-employed or retired individuals; §The Advanced Elderly Medical Service System for individuals aged ≥75 years

AIDS, autoimmune deficiency syndrome; COPD, chronic obstructive pulmonary disease; HIV, human immunodeficiency virus; SD, standard deviation

## Supplementary Table S6.

Average healthcare resource utilization events per season for influenza or its potential complications in each influenza season

|  | **Influenza season** | | | |
| --- | --- | --- | --- | --- |
|  | **2015/2016**  **N=909,104** | **2016/2017**  **N=2,060,597** | **2017/2018**  **N=2,374,679** | **2018/2019**  **N=3,630,328** |
| **ER visits for influenza†‡** |  |  |  |  |
| Absolute number of ER visits | 18 | 247 | 323 | 954 |
| Incidence proportion per 100,000 individuals | 1.98 | 11.99 | 13.60 | 26.28 |
| [95% CI] | [1.07–2.89] | [10.49–13.48] | [12.12–15.09] | [24.61–27.95] |
| **Outpatient visits for influenza†‡** |  |  |  |  |
| Absolute number of outpatient visits | 27**,**886 | 71**,**435 | 112**,**801 | 137**,**311 |
| Incidence proportion per 100,000 individuals | 3,067.42 | 3**,**466.71 | 4**,**750.16 | 3**,**782.33 |
| [95% CI] | [3**,**031.97–3**,**102.86] | [3**,**441.74–3**,**491.69] | [4**,**723.10–4**,**777.21] | [3**,**762.71–3**,**801.95] |
| **Hospitalizations for influenza†‡** |  |  |  |  |
| Absolute number of hospitalizations | 382 | 3,434 | 4,329 | 8,144 |
| Incidence proportion per 100,000 individuals | 42.02 | 166.65 | 182.30 | 224.33 |
| [95% CI] | [37.81–46.23] | [161.08–172.22] | [176.87–187.72] | [219.47–229.20] |
| **Hospitalizations for influenza broadly defined (influenza or pneumonia)‡§** | |  |  |  |
| Absolute number of hospitalizations | 6,492 | 26,216 | 26,979 | 51,824 |
| Incidence proportion per 100,000 individuals | 714.11 | 1,272.25 | 1,136.11 | 1,427.53 |
| [95% CI] | [696.80–731.42] | [1,256.95–1,287.55] | [1,122.63–1,149.59] | [1,415.33–1,439.73] |
| **Hospitalizations for respiratory disease‡¶** |  |  |  |  |
| Absolute number of hospitalizations | 11,967 | 45,056 | 47,970 | 90,341 |
| Incidence proportion per 100,000 individuals | 1,316.35 | 2,186.55 | 2,020.06 | 2,488.51 |
| [95% CI] | [1,292.92–1,339.78] | [2,166.58–2,206.52] | [2,002.17–2,037.96] | [2,472.48–2,504.53] |
| **Hospitalizations for respiratory or cardiovascular disease‡#** | |  |  |  |
| Absolute number of hospitalizations | 26,472 | 94,845 | 103,431 | 197,120 |
| Incidence proportion per 100,000 individuals | 2,911.88 | 4,602.79 | 4,355.58 | 5,429.81 |
| [95% CI] | [2,877.32–2,946.44] | [4,574.18–4,631.40] | [4,329.62–4,381.54] | [5406.50–5453.12] |
| **All-cause hospitalizations within 28 days of first influenza diagnosis†‡** | |  |  |  |
| Absolute number (all cases) of hospitalizations | 944 | 5,770 | 7,611 | 13,262 |
| Incidence per 100,000 individuals | 103.84 | 280.02 | 320.51 | 365.31 |
| [95% CI] | [97.22–110.46] | [272.80–287.23] | [313.32–327.70] | [359.11–371.52] |

†Defined as ICD-10 diagnostic codes J09–J11; ‡If an individual had multiple influenza-related ER visits or outpatient visits or was hospitalized multiple times for influenza or its potential complications within the influenza season, these were considered to be the same HCRU event; §Defined as ICD-10 diagnostic codes J09–J18; ¶Defined as ICD-10 diagnostic codes J09–J18, J40–J45, or J96; #Defined as ICD-10 diagnostic codes G45, G46, I20, I21, I24, I25, I50, I60–I63, I65, I66, I69, I67.8, I67.9, J09–J11, J40–J45, or J96

CI, confidence interval; ER, emergency room; HCRU, healthcare resource utilization; ICD-10, International Classification of Disease – 10^th^ revision; ICU, intensive care unit

## Supplementary Table S7.

Average healthcare resource utilization events per season (across all four seasons) for influenza or its potential complications in all individuals aged ≥60 to <65 years and in those with ≥1 comorbidity or without comorbidities

|  | **All individuals aged ≥60 to <65 years**  **N=1,299,552** | **Individuals aged ≥60 to <65 years** **with ≥1 comorbidity**  **N=440,350** | **Individuals aged ≥60 to <65 years** **without comorbidities**  **N=859,202** |
| --- | --- | --- | --- |
| **ER visits for influenza†‡** |  |  |  |
| Absolute number of ER visits | 63 | 25 | 38 |
| Incidence proportion per 100,000 individuals | 4.85 | 5.68 | 4.42 |
| [95% CI] | [3.65–6.04] | [3.45–7.90] | [3.02–5.83] |
| **Outpatient visits for influenza†‡** |  |  |  |
| Absolute number of outpatient visitis | 61,341 | 23,818 | 37,523 |
| Incidence proportion per 100,000 individuals | 4,720.17 | 5,408.88 | 4,367.19 |
| [95% CI] | [4,683.70–4,756.63] | [5,342.07–5,475.69] | [4,323.98–4,410.40] |
| **Hospitalizations for influenza†‡** |  |  |  |
| Absolute number of hospitalizations | 504 | 337 | 167 |
| Incidence proportion per 100,000 individuals | 38.78 | 76.53 | 19.44 |
| [95% CI] | [35.40–42.17] | [68.36–84.70] | [16.49–22.38] |
| **Hospitalizations for influenza broadly defined (influenza or pneumonia)‡§** |  |  |  |
| Absolute number of hospitalizations | 3,142 | 2,086 | 1,056 |
| Incidence proportion per 100,000 individuals | 241.78 | 473.71 | 122.90 |
| [95% CI] | [233.33–250.22] | [453.43–493.99] | [115.50–130.31] |
| **Hospitalizations for respiratory disease‡¶** |  |  |  |
| Absolute number of hospitalizations | 6,777 | 4,594 | 2,183 |
| Incidence proportion per 100,000 individuals | 521.49 | 1,043.26 | 254.07 |
| [95% CI] | [509.10–533.87] | [1,013.25–1,073.27] | [243.43–264.72] |
| **Hospitalizations for respiratory or cardiovascular disease‡#** |  |  |  |
| Absolute number of hospitalizations | 17,653 | 11,976 | 5,677 |
| Incidence proportion per 100,000 individuals | 1,358.39 | 2,719.65 | 660.73 |
| [95% CI] | [1,338.49–1,378.29] | [2,671.61–2,767.70] | [643.60–677.86] |
| **All-cause hospitalizations within 28 days of first influenza diagnosis†‡** |  |  |  |
| Absolute number of hospitalizations | 1,161 | 753 | 408 |
| Incidence proportion per 100,000 individuals | 89.34 | 171.00 | 47.49 |
| [95% CI] | [84.20–94.48] | [158.80–183.20] | [42.88–52.09] |

†Defined as ICD-10 diagnostic codes J09–J11; ‡If an individual had multiple influenza-related ER visits or outpatient visits or was hospitalized multiple times for influenza or its potential complications within the influenza season, these were considered to be the same HCRU event; §Defined as ICD-10 diagnostic codes J09–J18; ¶Defined as ICD-10 diagnostic codes J09–J18, J40–J45, or J96; #Defined as ICD-10 diagnostic codes G45, G46, I20, I21, I24, I25, I50, I60–I63, I65, I66, I69, I67.8, I67.9, J09–J11, J40–J45, or J96

CI, confidence interval; ER, emergency room; HCRU, healthcare resource utilization; ICD-10, International Classification of Disease – 10^th^ revision; ICU, intensive care unit; PY, person-years

## Supplementary Table S8.

Probability of inpatient mortality within 60 days of hospitalization for influenza or its potential complications (sensitivity analysis)

|  | **All individuals with  mortality data**  **N=1,758,000** | **Age group, years** | | |
| --- | --- | --- | --- | --- |
|  |  | **≥60 to <65**  **n=192,636** | **≥65 to <75**  **n=497,546** | **≥75**  **n=1,067,818** |
| **Hospitalizations for influenza†‡** | n=3,971 | n=76 | n=309 | n=3,586 |
| Absolute number of deaths | 402 | 4 | 33 | 365 |
| Probability of 60-day inpatient mortality, % | 10.12 | 5.26 | 10.68 | 10.18 |
| [95% CI] | [9.19–11.06] | [0.24–10.28] | [7.24–14.12] | [9.19–11.17] |
| **Hospitalizations for influenza broadly defined (influenza or pneumonia)‡§** | n=26,341 | n=463 | n=2,292 | n=23,586 |
| Absolute number of deaths | 4,930 | 60 | 430 | 4,440 |
| Probability of 60-day inpatient mortality, % | 18.72 | 12.96 | 18.76 | 18.82 |
| [95% CI] | [18.25–19.19] | [9.90–16.02] | [17.16–20.36] | [18.33–19.32] |
| **Hospitalizations for respiratory disease‡¶** | n=46,336 | n=1,023 | n=5,003 | n=40,310 |
| Absolute number of deaths | 8,661 | 128 | 916 | 7,617 |
| Probability of 60-day inpatient mortality, % | 18.69 | 12.51 | 18.31 | 18.90 |
| [95% CI] | [18.34–19.05] | [10.48–14.54] | [17.24–19.38] | [18.51–19.28] |
| **Hospitalizations for respiratory or cardiovascular disease‡#** | n=102,004 | n=2,619 | n=11,986 | n=87,399 |
| Absolute number of deaths | 12,418 | 184 | 1,319 | 10,915 |
| Probability of 60-day inpatient mortality, % | 12.17 | 7.03 | 11.00 | 12.49 |
| [95% CI] | [11.97–12.37] | [6.05–8.00] | [10.44–11.56] | [12.27–12.71] |
| **All-cause hospitalizations within 28 days of first influenza diagnosis†‡** | n=6,414 | n=165 | n=626 | n=5,623 |
| Absolute number of deaths | 695 | 7 | 64 | 624 |
| Probability of 60-day inpatient mortality, % | 10.84 | 4.24 | 10.22 | 11.10 |
| [95% CI] | [10.07–11.60] | [1.17–7.32] | [7.85–12.60] | [10.28–11.92] |

†Defined as ICD-10 diagnostic codes J09–J11; ‡If an individual had multiple influenza-related ER visits or outpatient visits or was hospitalized multiple times for influenza or its potential complications within the influenza season, these were considered to be the same HCRU event; §Defined as ICD-10 diagnostic codes J09–J18; ¶Defined as ICD-10 diagnostic codes J09–J18, J40–J45, or J96; #Defined as ICD-10 diagnostic codes G45, G46, I20, I21, I24, I25, I50, I60–I63, I65, I66, I69, I67.8, I67.9, J09–J11, J40–J45, or J96

CI, confidence interval; HCRU, healthcare resource utilization; ICD-10, International Classification of Disease – 10^th^ revision

## Supplementary Table S9.

Probability of inpatient mortality within 90 days of hospitalization for influenza or its potential complications (sensitivity analysis)

|  | **All individuals with mortality data**  **N=1,758,000** | **Age group, years** | | | |
| --- | --- | --- | --- | --- | --- |
|  |  | **≥60 to <65**  **n=192,636** | **≥65 to <75**  **n=497,546** | **≥75**  **n=1,067,818** |  |
| **Hospitalizations for influenza†‡** | n=3,971 | n=76 | n=309 | n=3,586 | |
| Absolute number of deaths | 484 | 5 | 42 | 437 | |
| Probability of 90-day inpatient mortality, % | 12.19 | 6.58 | 13.59 | 12.19 | |
| [95% CI] | [11.17–13.21] | [1.01–12.15] | [9.77–17.41] | [11.12–13.26] | |
| **Hospitalizations for influenza broadly defined (influenza or pneumonia)‡§** | n=26,341 | n=463 | n=2,292 | n=23,586 | |
| Absolute number of deaths | 5,870 | 75 | 499 | 5,296 | |
| Probability of 90-day inpatient mortality, % | 22.28 | 16.20 | 21.77 | 22.45 | |
| [95% CI] | [21.78–22.79] | [12.84–19.55] | [20.08–23.46] | [21.92–22.99] | |
| **Hospitalizations for respiratory disease‡¶** | n=46,336 | n=1,023 | n=5,003 | n=40,310 | |
| Absolute number of deaths | 10,209 | 153 | 1,052 | 9,004 | |
| Probability of 90-day inpatient mortality, % | 22.03 | 14.96 | 21.03 | 22.34 | |
| [95% CI] | [21.66–22.41] | [12.77–17.14] | [19.90–22.16] | [21.93–22.74] | |
| **Hospitalizations for respiratory or cardiovascular disease‡#** | n=102,004 | n=2,619 | n=11,986 | n=87,399 | |
| Absolute number of deaths | 14,910 | 226 | 1,509 | 13,175 | |
| Probability of 90-day inpatient mortality, % | 14.62 | 8.63 | 12.59 | 15.07 | |
| [95% CI] | [14.40–14.83] | [7.55–9.70] | [12.00–13.18] | [14.84–15.31] | |
| **All-cause hospitalizations within 28 days of first influenza diagnosis†‡** | n=6,414 | n=165 | n=626 | n=5,623 | |
| Absolute number of deaths | 841 | 9 | 76 | 756 | |
| Probability of 90-day inpatient mortality, % | 13.11 | 5.45 | 12.14 | 13.44 | |
| [95% CI] | [12.29–13.94] | [1.99–8.92] | [9.58–14.70] | [12.55–14.34] | |

†Defined as ICD-10 diagnostic codes J09–J11; ‡If an individual had multiple influenza-related ER visits or outpatient visits or was hospitalized multiple times for influenza or its potential complications within the influenza season, it was considered to be the same HCRU event; §Defined as ICD-10 diagnostic codes J09–J18; ¶Defined as ICD-10 diagnostic codes J09–J18, J40–J45, or J96; #Defined as ICD-10 diagnostic codes G45, G46, I20, I21, I24, I25, I50, I60–I63, I65, I66, I69, I67.8, I67.9, J09–J11, J40–J45, or J96

CI, confidence interval; HCRU, healthcare resource utilization; ICD-10, International Classification of Disease – 10^th^ revision; ICU, intensive care unit

## Supplementary Table S10.

Probability of inpatient mortality within 30 days of hospitalization due to influenza or its potential complications in each influenza season among individuals with available mortality data

|  | **Influenza season** | | | |
| --- | --- | --- | --- | --- |
|  | **2015/2016**  **N=127,786** | **2016/2017**  **N=139,344** | **2017/2018**  **N=198,235** | **2018/2019**  **N=1,292,635** |
| **Hospitalizations for influenza†‡** | n=29 | n=71 | n=121 | n=3,750 |
| Absolute number of deaths | 4 | 3 | 11 | 256 |
| Probability of 30-day inpatient mortality, % | 13.79 | 4.23 | 9.09 | 6.83 |
| [95% CI] | [1.24–26.34] | [0.00–8.90] | [3.97–14.21] | [6.02–7.63] |
| **Hospitalizations for influenza broadly defined (influenza or pneumonia)‡§** | n=460 | n=527 | n=703 | n=24,651 |
| Absolute number of deaths | 85 | 69 | 106 | 3,378 |
| Probability of 30-day inpatient mortality, % | 18.48 | 13.09 | 15.08 | 13.70 |
| [95% CI] | [14.93–22.03] | [10.21–15.97] | [12.43–17.72] | [13.27–14.13] |
| **Hospitalizations for respiratory disease‡¶** | n=1,041 | n=1,073 | n=1,623 | n=42,599 |
| Absolute number of deaths | 173 | 139 | 242 | 5,996 |
| Probability of 30-day inpatient mortality, % | 16.62 | 12.95 | 14.91 | 14.08 |
| [95% CI] | [14.36–18.88] | [10.95–14.96] | [13.18–16.64] | [13.75–14.41] |
| **Hospitalizations for respiratory or cardiovascular disease‡#** | n=2,399 | n=2,561 | n=3,983 | n=93,061 |
| Absolute number of deaths | 242 | 214 | 332 | 8,416 |
| Probability of 30-day inpatient mortality, % | 10.09 | 8.36 | 8.34 | 9.04 |
| [95% CI] | [8.88–11.29] | [7.28–9.43] | [7.48–9.19] | [8.86–9.23] |
| **All-cause hospitalizations within 28 days of first influenza diagnosis†‡** | n=95 | n=142 | n=239 | n=5,938 |
| Absolute number of deaths | 11 | 9 | 17 | 453 |
| Probability of 30-day inpatient mortality, % | 11.58 | 6.34 | 7.11 | 7.63 |
| [95% CI] | [5.14–18.01] | [2.33–10.35] | [3.85–10.37] | [6.95–8.30] |

†Defined as ICD-10 diagnostic codes J09–J11; ‡If an individual had multiple influenza-related ER visits or outpatient visits or was hospitalized multiple times for influenza or its potential complications within the influenza season, these were considered to be the same HCRU event; §Defined as ICD-10 diagnostic codes J09–J18; ¶Defined as ICD-10 diagnostic codes J09–J18, J40–J45, or J96; #Defined as ICD-10 diagnostic codes G45, G46, I20, I21, I24, I25, I50, I60–I63, I65, I66, I69, I67.8, I67.9, J09–J11, J40–J45, or J96

CI, confidence interval; HCRU, healthcare resource utilization; ICD-10, International Classification of Disease – 10^th^ revision

## Supplementary Table S11.

Probability of inpatient mortality following hospitalization due to influenza or its potential complications in all individuals aged ≥60 to <65 years and in those with or without ≥1 comorbidity among individuals with available mortality data

|  | **All individuals aged ≥60 to <65 years**  **N=192,636** | **Individuals aged ≥60 to <65 years with ≥1 comorbidity**  **N=67,323** | **Individuals aged ≥60 to <65 years without comorbidities**  **N=125,313** |
| --- | --- | --- | --- |
| **Hospitalizations for influenza†‡** | n=76 | n=53 |  |
| Absolute number of deaths within 30 days | 3 | 2 |  |
| Probability of 30-day inpatient mortality, % | 3.95 | 3.77 |  |
| [95% CI] | [0.00–8.33] | [0.00–8.90] |  |
| Absolute number of deaths within 60 days | 4 | 3 |  |
| Probability of 60-day inpatient mortality, % | 5.26 | 5.66 |  |
| [95% CI] | [0.24–10.28] | [0.00–11.88] |  |
| Absolute number of deaths within 90 days | 5 | 4 |  |
| Probability of 90-day inpatient mortality, % | 6.58 | 7.55 |  |
| [95% CI] | [1.01–12.15] | [0.44–14.66] |  |
| **Hospitalizations for influenza broadly defined (influenza or pneumonia)‡§** | n=463 | n=301 |  |
| Absolute number of deaths within 30 days | 51 | 36 |  |
| Probability of 30-day inpatient mortality, % | 11.02 | 11.96 |  |
| [95% CI] | [8.16–13.87] | [8.29–15.63] |  |
| Absolute number of deaths within 60 days | 60 | 43 |  |
| Probability of 60-day inpatient mortality, % | 12.96 | 14.29 |  |
| [95% CI] | [9.90–16.02] | [10.33–18.24] |  |
| Absolute number of deaths within 90 days | 75 | 55 |  |
| Probability of 90-day inpatient mortality, % | 16.20 | 18.27 |  |
| [95% CI] | [12.84–19.55] | [13.91–22.64] |  |
| **Hospitalizations for respiratory disease‡¶** | n=1,023 | n=686 |  |
| Absolute number of deaths within 30 days | 108 | 74 |  |
| Probability of 30-day inpatient mortality, % | 10.56 | 10.79 |  |
| [95% CI] | [8.67–12.44] | [8.47–13.11] |  |
| Absolute number of deaths within 60 days | 128 | 89 |  |
| Probability of 60-day inpatient mortality, % | 12.51 | 12.97 |  |
| [95% CI] | [10.48–14.54] | [10.46–15.49] |  |
| Absolute number of deaths within 90 days | 153 | 106 |  |
| Probability of 90-day inpatient mortality, % | 14.96 | 15.45 |  |
| [95% CI] | [12.77–17.14] | [12.75–18.16] |  |
| **Hospitalizations for respiratory or cardiovascular disease‡#** | n=2,619 | n=1,770 |  |
| Absolute number of deaths within 30 days | 153 | 93 |  |
| Probability of 30-day inpatient mortality, % | 5.84 | 5.25 |  |
| [95% CI] | [4.94–6.74] | [4.21–6.29] |  |
| Absolute number of deaths within 60 days | 184 | 119 |  |
| Probability of 60-day inpatient mortality, % | 7.03 | 6.72 |  |
| [95% CI] | [6.05–8.00] | [5.56–7.89] |  |
| Absolute number of deaths within 90 days | 226 | 151 |  |
| Probability of 90-day inpatient mortality, % | 8.63 | 8.53 |  |
| [95% CI] | [7.55–9.70] | [7.23–9.83] |  |
| **All-cause hospitalizations within 28 days of first influenza diagnosis†‡** | n=165 | n=113 |  |
| Absolute number of deaths within 30 days | 5 | 4 |  |
| Probability of 30-day inpatient mortality, % | 3.03 | 3.54 |  |
| [95% CI] | [0.41–5.65] | [0.13–6.95] |  |
| Absolute number of deaths within 60 days | 7 | 6 |  |
| Probability of 60-day inpatient mortality, % | 4.24 | 5.31 |  |
| [95% CI] | [1.17–7.32] | [1.18–9.44] |  |
| Absolute number of deaths within 90 days | 9 | 8 |  |
| Probability of 90-day inpatient mortality, % | 5.45 | 7.08 |  |
| [95% CI] | [1.99–8.92] | [2.35–11.81] |  |

†Defined as ICD-10 diagnostic codes J09–J11; ‡If an individual had multiple influenza-related ER visits or outpatient visits or was hospitalized multiple times for influenza or its potential complications within the influenza season, these were considered to be the same HCRU event; §Defined as ICD-10 diagnostic codes J09–J18; ¶Defined as ICD-10 diagnostic codes J09–J18, J40–J45, or J96; #Defined as ICD-10 diagnostic codes G45, G46, I20, I21, I24, I25, I50, I60–I63, I65, I66, I69, I67.8, I67.9, J09–J11, J40–J45, or J96

CI, confidence interval; HCRU, healthcare resource utilization; ICD-10, International Classification of Disease – 10^th^ revision

## Supplementary Table S12.

Median direct medical costs associated with healthcare resource utilization for influenza or its potential complications

| **Median (Q1–Q3) medical costs, ¥** | **Total study population†**  **N=8,974,708** | **Age group, years** | | |
| --- | --- | --- | --- | --- |
|  |  | **≥60 to <65**  **n=1,299,552** | **≥65 to <75**  **n=3,501,469** | **≥75**  **n=4,173,687** |
| **Total costs per event** |  |  |  |  |
| ER visits for influenza‡§ | 37,996.3  (27,086.9–51,715.0) | 37,546.0  (23,298.6–50,269.0) | 38,194.1  (26,694.3–51,721.1) | 37,966.0  (27,426.0–51,970.9) |
| Outpatient visits for influenza‡§ | 11,479.4  (9,511.0–14,141.3) | 11,176.5  (9,411.0–12,830.0) | 11,317.6  (9,477.0–13,298.6) | 11,967.0  (9,626.5–16,048.0) |
| **60-day costs per hospitalization** |  |  |  |  |
| Hospitalizations for influenza‡§ | 402,027.0  (188,000.0–961,785.6) | 395,269.8  (177,417.9–1,035,797.0) | 459,943.3  (183,556.2–1,081,288.7) | 395,984.2  (188,909.2–938,800.8) |
| Hospitalizations for influenza broadly defined (influenza or pneumonia)§¶ | 539,795.8  (253,519.8–1,104,897.8) | 483,775.6  (214,027.9–1,164,399.5) | 528,202.5  (232,129.8–1,177,007.0) | 543,763.4  (258,739.3–1,093,025.2) |
| Hospitalization for respiratory disease§# | 582,357.4  (263,111.5–1,182,373.1) | 575,537.5  (233,125.5–1,324,815.9) | 590,975.0  (248,348.3–1,310,165.8) | 580,808.8  (267,479.4–1,157,022.3) |
| Hospitalizations for respiratory or cardiovascular disease§†† | 611,006.4  (262,263.9–1,243,267.1) | 577,152.4  (223,713.2–1,284,518.9) | 600,856.4  (237,036.7–1,300,917.1) | 615,403.5  (270,797.8–1,228,721.0) |
| All-cause hospitalizations within 28 days of first influenza diagnosis‡§ | 436,558.4  (198,318.5–1,007,919.8) | 399,500.1  (165,135.8–978,105.2) | 450,279.1  (184,518.0–1,068,533.0) | 436,152.2  (201,645.9–999,790.1) |

†Individuals with missing medical cost data were excluded from this analysis; ‡Defined as ICD-10 diagnostic codes J09–J11; §If an individual had multiple influenza-related ER visits or outpatient visits or was hospitalized multiple times for influenza or its potential complications within the influenza season, these were considered to be the same HCRU event; ¶Defined as ICD-10 diagnostic codes J09–J18; #Defined as ICD-10 diagnostic codes J09–J18, J40–J45, or J96; ††Defined as ICD-10 diagnostic codes G45, G46, I20, I21, I24, I25, I50, I60–I63, I65, I66, I69, I67.8, I67.9, J09–J11, J40–J45, or J96

ER, emergency room; HCRU, healthcare resource utilizationICD-10, International Classification of Disease – 10^th^ revision; ICU, intensive care unit; Q1, first quartile; Q3, third quartile; ¥, Yen

## Supplementary Table S13.

Mean 30-day direct medical costs associated with hospitalization for influenza or its potential complications (sensitivity analysis)

| **Mean ± SD 30-day medical costs per event, ¥** | **Total study population†**  **N=8,974,708** | **Age group, years** | | |
| --- | --- | --- | --- | --- |
|  |  | **≥60 to <65**  **n=1,299,552** | **≥65 to <75**  **n=3,501,469** | **≥75**  **n=4,173,687** |
| Hospitalizations for influenza‡§ | 488,620.1 ± 692,003.4 | 618,799.5 ± 875,549.5 | 616,151.6 ± 1,362,147.1 | 461,737.0 ± 475,982.0 |
| Hospitalizations for influenza broadly defined (influenza or pneumonia)§¶ | 578,904.3 ± 678,850.7 | 718,375.3 ± 1,074,141.9 | 670,350.2 ± 978,592.1 | 557,330.3 ± 585,837.9 |
| Hospitalizations for respiratory disease§# | 634,269.8 ± 752,488.6 | 791,948.0,± 1,037,908.1 | 762,838.0 ± 1,040,986.7 | 598,291.1 ± 648,672.9 |
| Hospitalizations for respiratory or cardiovascular disease§^,^†† | 662,889.2 ± 777,312.3 | 781,372.3 ± 1,005,373.2 | 768,144.4 ± 1,012,709.7 | 628,524.8 ± 682,279.3 |
| All-cause hospitalizations within 28 days of first influenza diagnosis‡§ | 520,152.7 ± 669,741.3 | 617,907.2 ± 937,545.7 | 617,824.5 ± 1,099,679.5 | 493,729.8 ± 507,197.9 |

†Individuals with missing medical cost data were excluded from this analysis; ‡Defined as ICD-10 diagnostic codes J09–J11; §If an individual had multiple influenza-related ER visits or outpatient visits or was hospitalized multiple times for influenza or its potential complications within the influenza season, these were considered to be the same HCRU event; ¶Defined as ICD-10 diagnostic codes J09–J18; #Defined as ICD-10 diagnostic codes J09–J18, J40–J45, or J96; ††Defined as ICD-10 diagnostic codes G45, G46, I20, I21, I24, I25, I50, I60–I63, I65, I66, I69, I67.8, I67.9, J09–J11, J40–J45, or J96

HCRU, healthcare resource utilization; ICD-10, International Classification of Disease – 10^th^ revision; ICU, intensive care unit; SD, standard deviation; ¥, Yen

## Supplementary Table S14.

Mean 90-day direct medical costs associated with hospitalization for influenza or its potential complications (sensitivity analysis)

| **Mean ± SD 90-day medical costs per event, ¥** | **Total study population†**  **N=8,974,708** | **Age group, years** | | |
| --- | --- | --- | --- | --- |
|  |  | **≥60 to <65**  **n=1,299,552** | **≥65 to <75**  **n=3,501,469** | **≥75**  **n=4,173,687** |
| Hospitalizations for influenza‡§ | 818,953.5 ± 1,084,542.1 | 948,448.0 ± 1,275,118.2 | 973,319.4 ± 1,847,740.0 | 787,454.3 ± 876,148.3 |
| Hospitalizations for influenza broadly defined (influenza or pneumonia)§¶ | 951,727.2 ± 1,083,264.5 | 1,105,871.2 ± 1,540,668.1 | 1,074,044.7 ± 1,462,058.3 | 923,969.5 ± 975,134.5 |
| Hospitalizations for respiratory disease§# | 1,017,093.9 ± 1,138,703.2 | 1,177,963.5 ± 1,482,877.9 | 1,156,623.3 ± 1,469,768.9 | 978,482.8 ± 1,027,027.1 |
| Hospitalizations for respiratory or cardiovascular disease§†† | 1,064,300.6 ± 1,172,532.1 | 1,145,469.7 ± 1,407,502.1 | 1,153,520.4 ± 1,420,816.0 | 1,036,224.8 ± 1,080,665.6 |
| All-cause hospitalizations within 28 days of first influenza diagnosis‡§ | 854,788.9 ± 1,076,943.8 | 896,747.5 ± 1,307,003.9 | 947,307.4 ± 1,582,336.6 | 832,465.3 ± 914,610.9 |

†Individuals with missing medical cost data were excluded from this analysis; ‡Defined as ICD-10 diagnostic codes J09–J11; §If an individual had multiple influenza-related ER visits or outpatient visits or was hospitalized multiple times for influenza or its potential complications within the influenza season, these were considered to be the same HCRU event; ¶Defined as ICD-10 diagnostic codes J09–J18; #Defined as ICD-10 diagnostic codes J09–J18, J40–J45, or J96; ††Defined as ICD-10 diagnostic codes G45, G46, I20, I21, I24, I25, I50, I60–I63, I65, I66, I69, I67.8, I67.9, J09–J11, J40–J45, or J96

HCRU, healthcare resource utilization; ICD-10, International Classification of Disease – 10^th^ revision; ICU, intensive care unit; SD, standard deviation; ¥, Yen

## Supplementary Table S15.

Mean direct medical costs associated with healthcare resource utilization for influenza or its potential complications in each individual influenza season

| **Mean ± SD medical costs, ¥** | **Influenza season** | | | |
| --- | --- | --- | --- | --- |
|  | **2015/2016**  **N=909,104** | **2016/2017**  **N=2,060,597** | **2017/2018**  **N=2,374,679** | **2018/2019**  **N=3,630,328** |
| **Total costs per event** |  |  |  |  |
| ER visits for influenza†‡ | 42,383.6 ± 15,660.8 | 41,580.0 ± 18,132.1 | 41,206.1 ± 15,590.9 | 40,713.6 ± 17,499.8 |
| Outpatient visits for influenza†‡ | 12,334.8 ± 14,977.6 | 13,553.7 ± 10,071.6 | 13,273.5 ± 10,270.6 | 14,137.1 ± 12,860.9 |
| **60-day costs per hospitalization** |  |  |  |  |
| Hospitalizations for influenza†‡ | 672,111.0 ± 886,090.9 | 642,850.3 ± 73,8131.4 | 668,990.4 ± 741,856.2 | 700,991.5 ± 1,034,735.1 |
| Hospitalization for influenza broadly defined (influenza or pneumonia)‡§ | 834,058.7 ± 945,069.6 | 756,864.7 ± 806,677.1 | 773,492.6 ± 863,675.8 | 821,929.8 ± 942,214.6 |
| Hospitalization for respiratory disease‡¶ | 899,935.2 ± 1,031,507.6 | 821,403.0 ± 910,861.8 | 840,303.3 ± 941,130.3 | 882,313.2 ± 984,912.7 |
| Hospitalization for respiratory or cardiovascular disease‡# | 922,814.9 ± 1,074,836.3 | 870,582.6 ± 954,353.6 | 890,792.8 ± 988,959.2 | 913,175.6 ± 996,616.9 |
| All-cause hospitalizations within 28 days of first influenza diagnosis†‡ | 749,548.1 ± 970,337.9 | 683,146.2 ± 776,326.5 | 705,928.1 ± 810,650.7 | 733293.0 ± 967268.0 |

†Defined as ICD-10 diagnostic codes J09–J11; ‡If an individual had multiple influenza-related ER visits or outpatient visits or was hospitalized multiple times for influenza or its potential complications within the influenza season, these were considered to be the same HCRU event; §Defined as ICD-10 diagnostic codes J09–J18; ¶Defined as ICD-10 diagnostic codes J09–J18, J40–J45, or J96; #Defined as ICD-10 diagnostic codes G45, G46, I20, I21, I24, I25, I50, I60–I63, I65, I66, I69, I67.8, I67.9, J09–J11, J40–J45, or J96

ER, emergency room; HCRU, healthcare resource utilization; ICD-10, International Classification of Disease – 10^th^ revision; ICU, intensive care unit; SD, standard deviation; ¥, Yen

## Supplementary Table S16.

Direct medical costs associated with healthcare resource utilization for influenza or its potential complications in all individuals aged ≥60 to <65 years and in those with ≥1 comorbidity or without comorbidities

| **Mean ± SD medical costs, ¥** | **All individuals aged ≥60 to <65 years**  **N=1,299,552** | **Individuals aged ≥60 to <65 years with ≥1 comorbidity**  **N=440,350** |  |
| --- | --- | --- | --- |
| **Total costs per event** |  |  |  |
| ER visits for influenza†‡ | 38,429.4 ± 16,175.1 | 38,533.9 ± 13,682.4 |  |
| Outpatient visits for influenza†‡ | 12,226.7 ± 12,270.7 | 13,287.5 ± 15,257.1 |  |
| **Hospitalization costs per hospitalization** |  |  |  |
| Hospitalizations for influenza†‡ |  |  |  |
| 30-day costs | 618,799.5 ± 875,549.5 | 561,941.7 ± 720,385.9 |  |
| 60-day costs | 817,528.1 ± 1,146,961.6 | 754,770.3 ± 1,016,525.5 |  |
| 90-day costs | 948,448.0 ± 1,275,118.2 | 876,459.0 ± 1,122,364.9 |  |
| Hospitalizations for influenza broadly defined (influenza or pneumonia)‡§ | | | |
| 30-day costs | 718,375.3 ± 1,074,141.9 | 702,316.3 ± 1,062,363.0 |  |
| 60-day costs | 945,445.1 ± 1,357,674.7 | 937,202.6 ± 1,351,317.0 |  |
| 90-day costs | 1,105,871.2 ± 1,540,668.1 | 1,105,960.8 ± 1,534,897.6 |  |
| Hospitalizations for respiratory disease‡¶ | | | |
| 30-day costs | 791,948.0,± 1,037,908.1 | 759,761.5 ± 977,917.8 |  |
| 60-day costs | 1,016,978.0 ± 1,294,437.8 | 992,682.5 ± 1,257,080.0 |  |
| 90-day costs | 1,177,963.5 ± 1,482,877.9 | 1,160,141.6 ± 1,457,992.0 |  |
| Hospitalizations for respiratory or cardiovascular disease‡# | | | |
| 30-day costs | 781,372.3 ± 1,005,373.2 | 743,265.5 ± 964,444.2 |  |
| 60-day costs | 989,030.9 ± 1,222,902.5 | 948,425.3 ± 1,196,077.8 |  |
| 90-day costs | 1,145,469.7 ± 1,407,502.1 | 1,103,247.0 ± 1,386,603.0 |  |
| All-cause hospitalizations within 28 days of first influenza diagnosis†‡ | | | |
| 30-day costs | 617,907.2 ± 937,545.7 | 615,544.8 ± 934,913.3 |  |
| 60-day costs | 779,163.4 ± 1,157,676.8 | 787,655.6 ± 1,173,660.0 |  |
| 90-day costs | 896,747.5 ± 1,307,003.9 | 913,711.4 ± 1,309,840.9 |  |

†Defined as ICD-10 diagnostic codes J09–J11; ‡If an individual had multiple influenza-related ER visits or outpatient visits or was hospitalized multiple times for influenza or its potential complications within the influenza season, it was considered to be the same HCRU event; §Defined as ICD-10 diagnostic codes J09–J18; ¶Defined as ICD-10 diagnostic codes J09–J18, J40–J45, or J96; #Defined as ICD-10 diagnostic codes G45, G46, I20, I21, I24, I25, I50, I60–I63, I65, I66, I69, I67.8, I67.9, J09–J11, J40–J45, or J96

ER, emergency room; HCRU, healthcare resource utilization; ICD-10, International Classification of Disease – 10^th^ revision; ICU, intensive care unit; SD, standard deviation; ¥, Yen

## Supplementary Figure S1.

Definitions of the four influenza seasons and associated baseline periods (i.e., before the start of each influenza season) during the study period


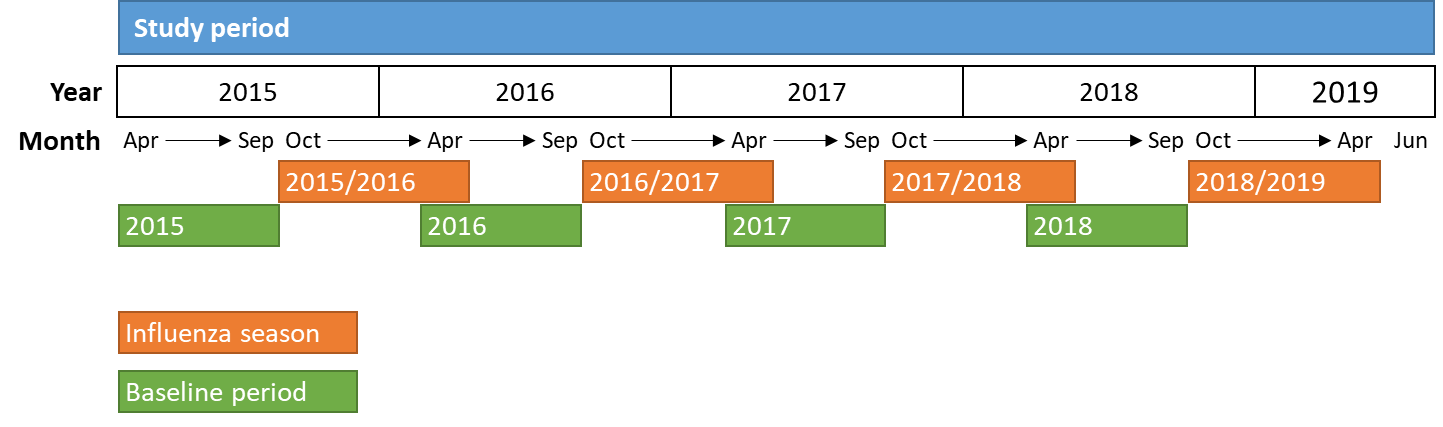


## Supplementary Figure S2.

Distribution of the duration for hospitalizations for (a) influenza†‡ (N=16,289), (b) influenza broadly defined (influenza or pneumonia)‡§ (N=111,511), (c) respiratory disease‡¶ (N=195,334), and (d) respiratory or cardiovascular disease‡# (N=421,868) across all four seasons in the total study population


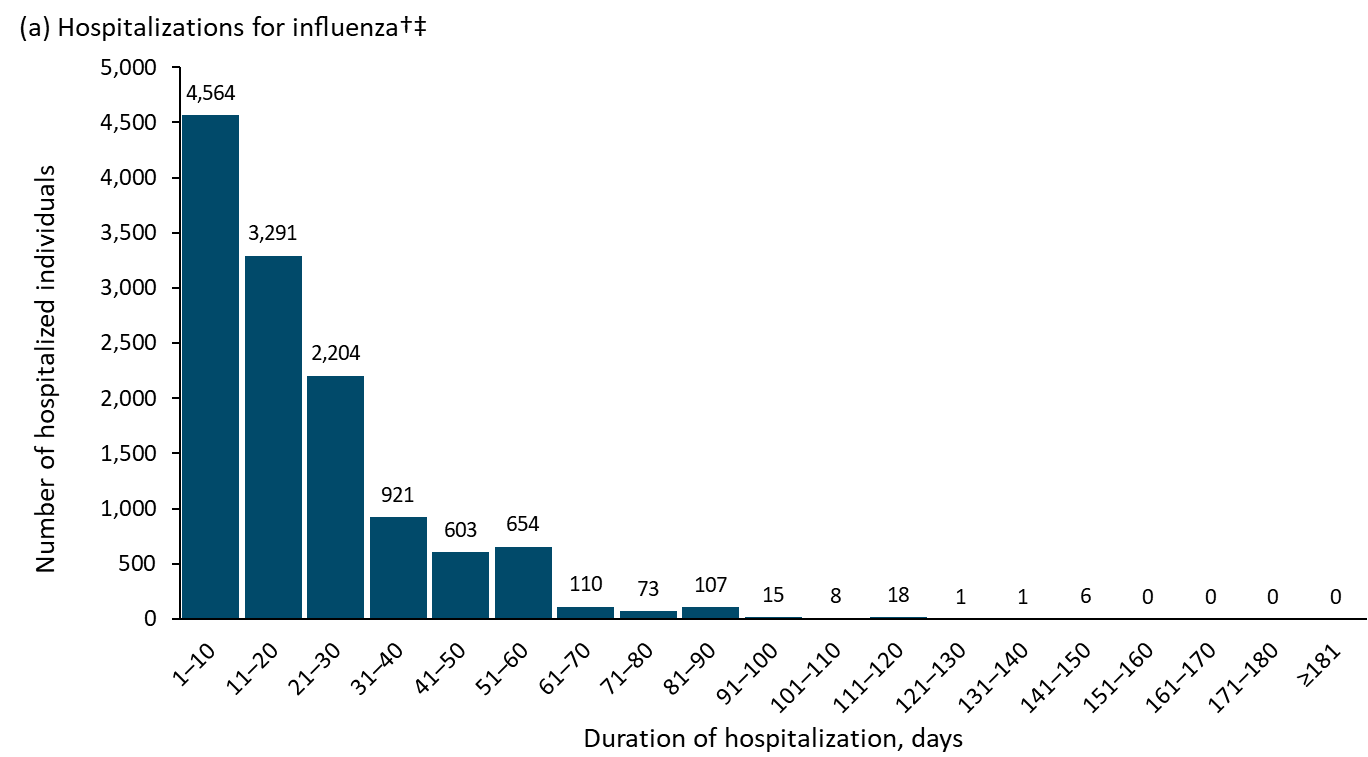


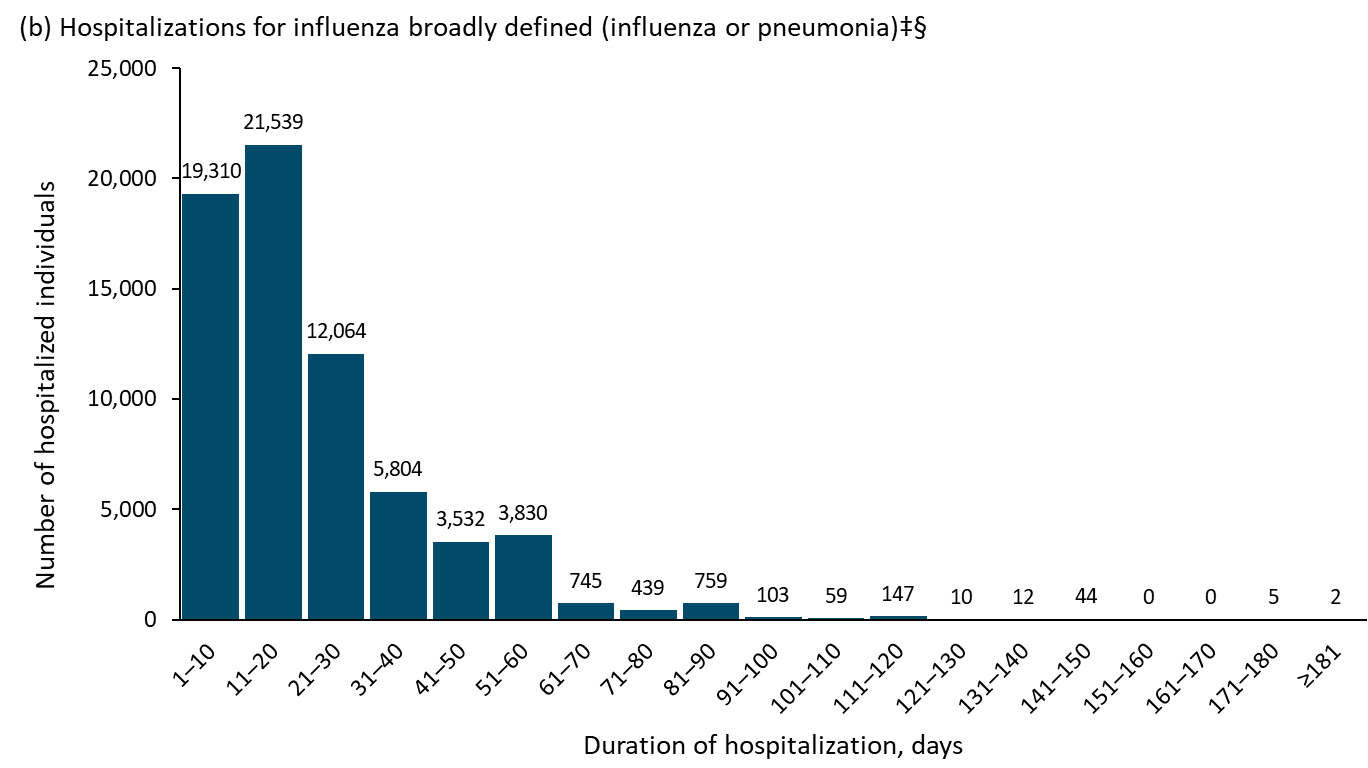


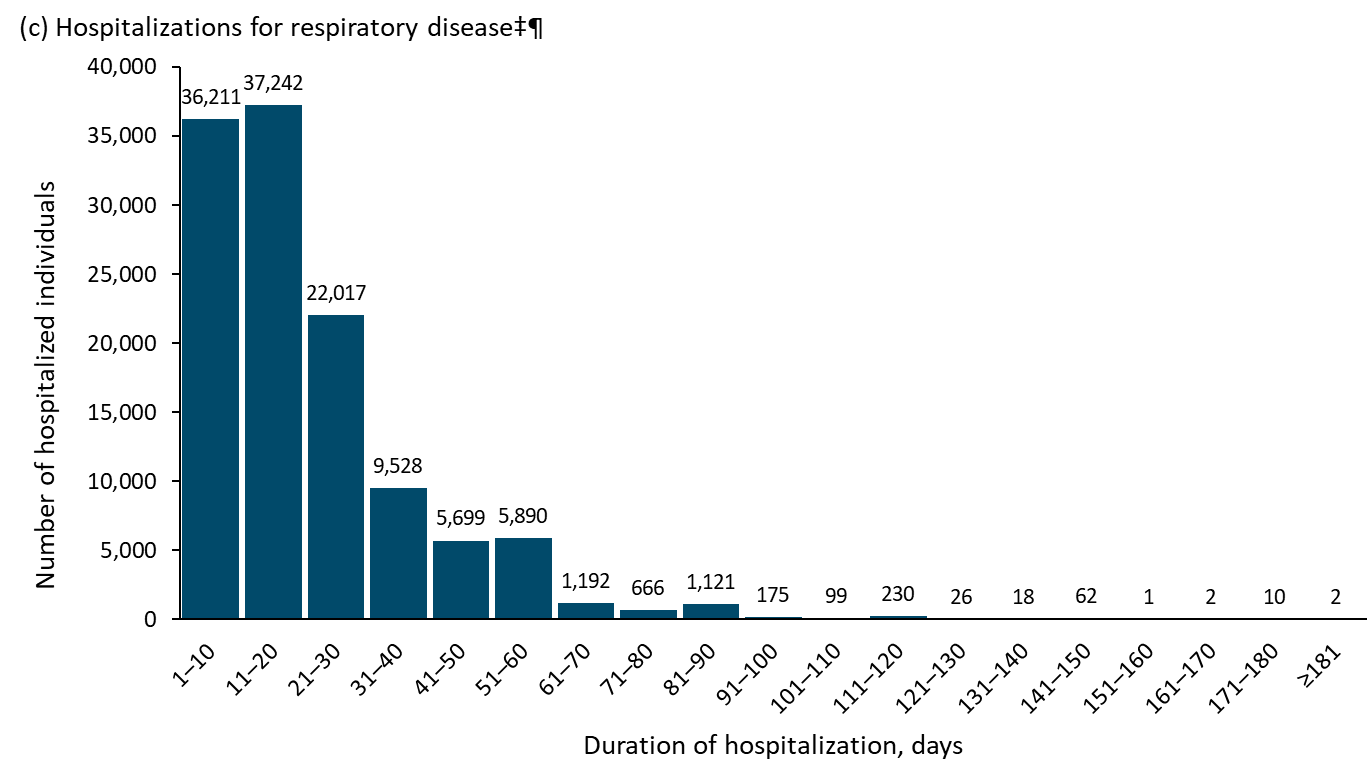


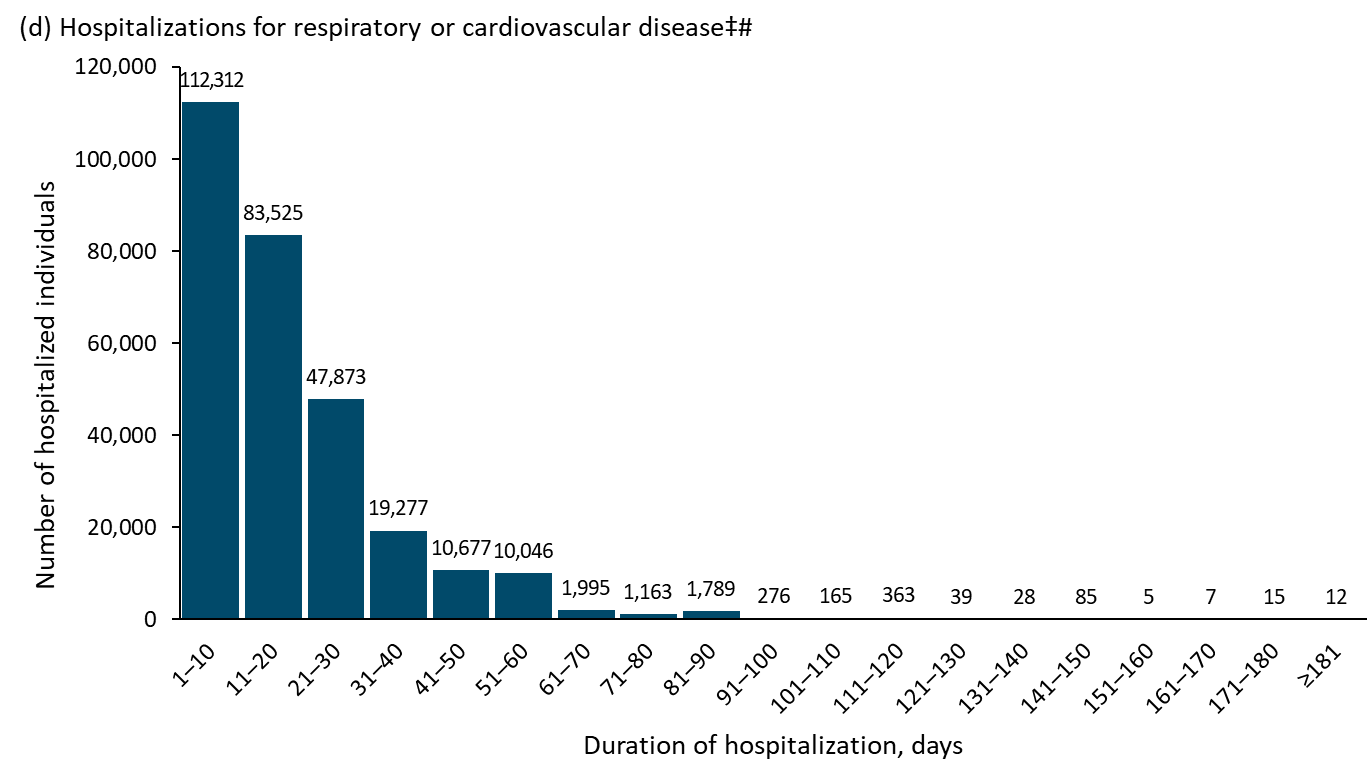


†Defined as International Classification of Disease – 10^th^ revision (ICD-10) diagnostic codes J09–J11; ‡If an individual had multiple influenza-related ER visits or outpatient visits or was hospitalized multiple times for influenza or its potential complications within the influenza season, these were considered to be the same HCRU event; §Defined as ICD-10 diagnostic codes J09–J18; ¶Defined as ICD-10 diagnostic codes J09–J18, J40–J45, or J96; #Defined as ICD-10 diagnostic codes G45, G46, I20, I21, I24, I25, I50, I60–I63, I65, I66, I69, I67.8, I67.9, J09–J11, J40–J45, or J96

HCRU, healthcare resource utilization

# References

1. DiazGranados CA, Robertson CA, Talbot HK, Landolfi V, Dunning AJ, Greenberg DP. Prevention of serious events in adults 65 years of age or older: a comparison between high-dose and standard-dose inactivated influenza vaccines. *Vaccine.* 2015;33(38):4988-4993.
